# Comprehensive Custom NGS Panel Validation for the Improvement of the Stratification of B-Acute Lymphoblastic Leukemia Patients

**DOI:** 10.3390/jpm10030137

**Published:** 2020-09-21

**Authors:** Adrián Montaño, Jesús Hernández-Sánchez, Maribel Forero-Castro, María Matorra-Miguel, Eva Lumbreras, Cristina Miguel, Sandra Santos, Valentina Ramírez-Maldonado, José Luís Fuster, Natalia de Las Heras, Alfonso García-de Coca, Magdalena Sierra, Julio Dávila, Ignacio de la Fuente, Carmen Olivier, Juan Olazabal, Joaquín Martínez, Nerea Vega-García, Teresa González, Jesús María Hernández-Rivas, Rocío Benito

**Affiliations:** 1 IBSAL, IBMCC, Universidad de Salamanca, CSIC, Centro de Investigación del Cáncer (CIC), 37007 Salamanca, Spain; adrianmo18@gmail.com (A.M.); jesus807@gmail.com (J.H.-S.); mariammzamora@gmail.com (M.M.-M.); a21093@usal.es (E.L.); cristinamiguelgarcia@gmail.com (C.M.); sandruskism90@gmail.com (S.S.); vramirem@gmail.com (V.R.-M.); 2 Escuela de Ciencias Biológicas (Grupo de investigación GICBUPTC), Universidad Pedagógica y Tecnológica de Colombia, Tunja 150003, Colombia; maribel.forero@uptc.edu.co; 3 Sección de Oncohematología Pediátrica, Hospital Clínico Universitario Virgen de la Arrixaca, Instituto Murciano de Investigación Biosanitaria (IMIB), 30120 Murcia, Spain; josel.fuster@carm.es; 4 Departamento de Hematología—Hospital Virgen Blanca, 24008 León, Spain; nherasr@saludcastillayleon.es; 5 Departamento de Hematología—Hospital Clínico de Valladolid, 47003 Valladolid, Spain; agarciaco@saludcastillayleon.es; 6 Complejo Sanitario de Zamora, 49022 Zamora, Spain; msierrap@saludcastillayleon.es; 7 Departamento de Hematología—Hospital Universitario de Salamanca, 37007 Salamanca, Spain; juldaval@hotmail.com (J.D.); teresa.gonzalez.mart@gmail.com (T.G.); 8 Departamento de Hematología—Hospital Rio Hortega, 47012 Valladolid, Spain; ifuentegr@saludcastillayleon.es; 9 Servicio de Hematología y Hemoterapia—Complejo Sanitario de Segovia, 40002 Segovia, Spain; colivierco@gmail.com; 10 Departamento de Hematología—Hospital Universitario de Burgos, 09006 Burgos, Spain; jolazabal@saludcastillayleon.es; 11 Departamento de Hematología—Hospital Universitario 12 de Octubre, 28041 Madrid, Spain; jmarti01@med.ucm.es; 12 Laboratorio de Hematología, Instituto de Investigación, Hospital Sant Joan de Déu, 08950 Barcelona, Spain; nvega@fsjd.org

**Keywords:** acute lymphoblastic leukemia, NGS, genetic alterations, diagnosis

## Abstract

Background: B-acute lymphoblastic leukemia (B-ALL) is a hematological neoplasm of the stem lymphoid cell of the B lineage, characterized by the presence of genetic alterations closely related to the course of the disease. The number of alterations identified in these patients grows as studies of the disease progress, but in clinical practice, the conventional techniques frequently used are only capable of detecting the most common alterations. However, techniques, such as next-generation sequencing (NGS), are being implemented to detect a wide spectrum of new alterations that also include point mutations. Methods: In this study, we designed and validated a comprehensive custom NGS panel to detect the main genetic alterations present in the disease in a single step. For this purpose, 75 B-ALL diagnosis samples from patients previously characterized by standard-of-care diagnostic techniques were sequenced. Results: The use of the custom NGS panel allowed the correct detection of the main genetic alterations present in B-ALL patients, including the presence of an aneuploid clone in 14 of the samples and some of the recurrent fusion genes in 35 of the samples. The panel was also able to successfully detect a number of secondary alterations, such as single nucleotide variants (SNVs) and copy number variations (CNVs) in 66 and 46 of the samples analyzed, respectively, allowing for further refinement of the stratification of patients. The custom NGS panel could also detect alterations with a high level of sensitivity and reproducibility when the findings obtained by NGS were compared with those obtained from other conventional techniques. Conclusions: The use of this custom NGS panel allows us to quickly and efficiently detect the main genetic alterations present in B-ALL patients in a single assay (SNVs and insertions/deletions (INDELs), recurrent fusion genes, CNVs, aneuploidies, and single nucleotide polymorphisms (SNPs) associated with pharmacogenetics). The application of this panel would thus allow us to speed up and simplify the molecular diagnosis of patients, helping patient stratification and management.

## 1. Introduction

B-acute lymphoblastic leukemia (B-ALL) is a malignancy of hematopoietic stem cells, which originates in the B-line lymphoid and is characterized by the presence of a series of genetic alterations, mainly translocations, which determine the course of the disease. Each alteration is associated with a low, intermediate, or high risk. The current WHO classification system is based on the presence or absence of these genetic alterations, which allows stratification of approximately 80% of children and 75% of adults with B-ALL [1,2]. However, there is still a high percentage of patients, commonly referred to as “B-other” cases, who do not have any of the recurrent genetic alterations and, therefore, cannot be stratified according to risk. There is also great clinical heterogeneity within each of the different prognostic subgroups [3,4].

In the last decade, technical advances in microarray and sequence analysis have allowed the development of multiple comprehensive exploratory studies, through which a number of candidate prognostic markers for ALL risk stratification have been identified and published. These alterations range from point mutations; changes in copy number, which affects key genes in B-ALL development, such as *IKZF1*, *PAX5*, *CDKN2A, ETV6, BTG1,* and *RB1*; new rearrangements, such as those affecting the *CRLF2* gene [2,5,6,7,8]. Some of them have already been considered as biological markers of poor prognosis, especially the loss of *IKZF1* [9]. In addition, a number of polymorphisms and mutations directly associated with the response to treatments have been identified [10,11,12,13]. Most of them are not yet considered in clinical practice, nor are they even routinely identified at diagnosis, although in the future, they are likely to contribute to the risk stratification of patients and be taken into account in therapeutic decisions [2,8,11]. 

Diagnostic techniques, such as karyotyping and fluorescence in situ hybridization (FISH), remain the gold standard in clinical practice in the diagnosis of B-ALL patients [14,15]. In addition, other conventional techniques, such as SNPs arrays and multiplex ligation-dependent probe amplification (MLPA), are often used to identify smaller CNVs and larger losses/gains [16]. However, these techniques are not always sufficient to identify the broad spectrum of new alterations detected in the disease and also have significant limitations in terms of detection sensitivity [9,17,18]. 

The development of mass sequencing techniques in recent years has allowed their implementation in the study of hematological malignancies, such as B-ALL. Consequently, they have become a promising tool for the clinical management of the disease. These techniques include the study of DNA, RNA, and miRNA and can involve a variety of strategies, including whole-genome sequencing (WGS), whole-exome sequencing (WES), and targeted panels. However, WGS and WES, which currently allow the detection of the largest number of alterations, remain expensive, slow, and labor-intensive in the clinical laboratory [19,20]. For this reason, in recent years, targeted panels have taken priority over WGS and WES [21,22]. This study, therefore, proposed the use of specific panels as the best option for clinical practice, through which detection sensitivity can be improved and data analysis simplified.

In this study, we proposed the use of a specific DNA-based panel to help overcome the limitations of the techniques conventionally used in clinical practice during the process of patient diagnosis. The incorporation of selective sequencing approaches in clinical practice could allow the single-step detection of the main genetic alterations present in B-ALL with greater sensitivity, in a fast, simple, and economic way. In addition, the applicability of this could be guaranteed since it is designed for use in bench-top sequencers. These alterations include: (a) aneuploidies, high hyperdiploid (>51 chromosomes), low hypodiploid (31-39 chromosomes), and near-haploid (24–30 chromosomes); (b) recurrent fusion genes (*ETV6/RUN*X1, *BCR/ABL1* and *MLL*r); (c) intrachromosomal amplification of chromosome 21 (iAMP21) and *CRLF2* rearrangements; (d) CNVs, in particular, alterations in *IKZF1*, *PAX5*, *CDKN2A*, *ETV6*, *BTG1*, and *RB1* genes; (e) candidate actionable genes, which can only be detected by NGS; (f) polymorphisms or germinal mutations associated with treatment response.

In this work, we designed a customized DNA-based NGS panel, whose validation by standard diagnostic tests is expected to allow us to use a unique methodology to detect the main genetic alterations present in B-ALL and to achieve a better stratification of patients according to risk. The study included 75 samples that had previously been characterized by conventional techniques. The use of the custom NGS panel would allow the correct stratification, as well as a refined prognosis of patients by a genuinely comprehensive assessment of the molecular spectrum of alterations (CNVs, nucleotide mutations, SNPs associated with pharmacogenetic and fusion genes), as well as cooperating genetic aberrations (e.g., *IKZF1, CDKN2A*, *PAX5*, *BTG1*, and *CRLF2* rearrangements). 

## 2. Materials and Methods

### 2.1. Selection of Patients and Samples

Seventy-five B-ALL patients at diagnosis referred from 22 Spanish centers to the Hematology Service at the University Hospital, Salamanca, were analyzed in this study. The diagnosis of B-ALL patients was based on the morphological, immunophenotypic, and genetic characteristics of leukemic blast cells, as described above [23]. Standard-of-care diagnostics also include genetic characterization by conventional cytogenetic analysis, karyotype, and FISH. 

The samples were also studied using other methodologies to validate them: 62 of the samples were sequenced using other sequencing methodologies; 44 were sequenced by 454 Junior sequencing (Roche, Basel, Switzerland); one was sequenced by an amplicon-based NGS panel (Illumina, San Diego, CA, USA); 17 samples were sequenced using both methodologies. The characteristics of these panels are detailed below in the section on validation assays.

Sixty-two of the 75 samples were also studied by MLPA (for *IKZF1*, *CDKN2A/B*, *PAX5*, *EBF1*, *ETV6*, *BTG1*, and *RB1* genes, as well as genes from the X/Y PAR1 region) and microarray-based comparative genomic hybridization (aCGH) [24]. The loss of *IKZF1* in these patients was also verified by PCR. 

The cohort of patients included: 10 *BCRL/ABL1*-positive patients; 10 *ETV6/RUNX1*-positive patients; 10 patients with a high hyperdiploid clone (>51 chromosomes); 10 patients with *MLL*r; 3 patients with a low hypodiploidy clone (<40 chromosomes); 3 patients with iAMP21, and 29 without recurrent disorders or B-other patients (two of whom had the *CRLF2* rearrangement). The characteristics of the patients are shown in Tables S1 and S2.

ALL cell lines were also used for technical validation. The REH cell line was obtained from Deutsche Sammlung von Mikroorganismen und Zellkulturen (DMSZ) German collection (ACC 22). It was established from the peripheral blood of a patient with ALL, who carried t (12;21) and del (12), producing respective *ETV6/RUNX1* fusion and deletion of *ETV6*. This cell line has a series of mutations described in previous studies, which are collated online at https://cansarblack.icr.ac.uk/cell-line/REH/mutations. In addition, different clones generated in the laboratory from the REH line by single-cell sorting were used, in which the expression of the fusion protein *ETV6/RUNX1* was truncated using the clustered regularly interspaced short palindromic repeats-cas9 (CRISPR/Cas9) system [25].

The study was approved by the local ethical committee, the Comité Ético de Investigación Clínica del Área de Salud de Salamanca, at the University Hospital, Salamanca (ethical approval code: PI 2019 03 230). Written informed consent was obtained from each patient or legal guardian before patients entered the study.

### 2.2. DNA Extraction

Genomic DNA was extracted from frozen bone marrow or fixed peripheral blood cell samples with a QIAmp DNA mini kit (Qiagen, Valencia, CA, USA), following the manufacturer’s recommendations. DNA quantity was assessed by Qubit 4.0 using a double-strand kit (high/broad sensitivity) (Invitrogen Life Technologies, Carlsbad, CA, USA). 

### 2.3. Next-Generation Sequencing

#### 2.3.1. Custom NGS Panel Design

A custom NGS panel was made using SureDesign Studio (Agilent Technologies, Santa Clara, CA, USA) with 57,680 probes targeting 1522 regions, including specific exons for 150 genes associated with B-ALL; 165 SNPs associated with the pharmacogenetics of ALL treatment, breakpoint sequence of *ETV6/RUNX1, BCR/ABL1*, *MLL*r, *CRLF2*r, including *CRLF2/P2YR8* and *IGH/CRLF2* fusion gene; specific regions for CNV detection in *IKZF1*, *CDKN2A*, *PAX5*, *BTG1*, *RB1,* and *ETV6;* probes randomly distributed between chromosomes 4, 8, 10, and 21 for hyperdiploidy detection, and chromosomes 7 and 17 for hypodiploidy detection. The final design covered ~500 kb (Tables S3–S7). 

#### 2.3.2. Library Construction and Illumina Sequencing

Genomic DNA from each sample was fragmented and used to prepare the NGS libraries for the Illumina platform using a hybridized capture-based target-enrichment approach (SureSelect) developed by Agilent Technologies. Regions of interest were enriched for each library using the SureSelectQXT Target Enrichment Kit following the manufacturer’s instructions (Agilent). Enriched DNA was precipitated with streptavidin-coated beads and washed, eluted, and amplified with index tags to identify each sample and pooled for sequencing. After quality control was measured by the 4200 TapeStation (Agilent) and quantified using Qubit^®^ 4.0 Fluorometer, the libraries were sequenced on an Illumina NextSeq or MiSeq platform (Illumina Inc., San Diego, CA, USA). Paired-end sequencing (151-bp reads) was run. At the end of the process, this platform collects all the information in demultiplexed and paired FASTQ files for subsequent bioinformatic analysis.

#### 2.3.3. Sequence Data Processing: Mutational Analysis

Raw data quality control was performed with FastQc (v0.11.8) and Picard tools (v2.2.4) to collect sequencing metrics. Clonal reads were removed, and low-quality sequence tails were trimmed, with a threshold Phred quality score of ten. The trimmed sequence reads were aligned to the reference genome (hg38/GRCh38) and to mark PCR duplicates, BWA v0.7.12, and GATK v3.5.

A minimum quality score of Q30 was required to ensure high-quality sequencing results. Variant calling and annotation were performed using an in-house pipeline based on the VarScan v2.3.9, SAMTools v1.3.1., and ANNOVAR bioinformatic tools. The dbSNP, COSMIC, ClinVar, 1000 Genomes Browser, and exome aggregation consortium (ExAC) databases and in silico predictors of functional effects, such as mutationAssessor, sorting intolerant from tolerant (SIFT), and polymorphism phenotyping v2 (PolyPhen-2), were used for annotation. General information about variants was obtained using the Varsome web tool (https://varsome.com) [26]. To consider SNPs, variants with a minor allele frequency (MAF) of <0.01 were selected. In addition, variants with a variant allele frequency (VAF) of 40–60% or >90% were reviewed, prioritizing variants described by in silico predictors of functional effects and ClinVar as deleterious, damaging, pathogenic, or likely pathogenic. Aligned reads were manually reviewed with the integrative genomics viewer (IGV) to confirm and interpret variant calls, fusion genes, and microdeletions and reduce the risk of false positives. Any mutation in driver genes previously described in seminal papers was considered in the analysis.

#### 2.3.4. Sequence Data Processing: Copy Number Variation Analysis

Data were processed using a previously described in-house pipeline [27]. First, a reference was generated with control samples (unaltered karyotype). To that end, the mean coverage depth of each individual target of a sample was first normalized for the total amount of the DNA template loaded onto sequencing flow cells based on the total reads of that sample. The mean coverage of each individual target from the reference samples obtained in this was used as the reference for a specific region. To detect CNVs, the normalized coverage of each target of a test sample was compared with the mean coverage of the same region in the reference file generated, as described above. Whole-genome profiles of log_2_ ratios of mean coverage of individual targets of a region normalized to that of the reference were plotted against the target. The x-axis shows the targets in the panel plotted by relative genome order. The y-axis corresponds to the log_2_ ratio of mean coverage of testing to that of the reference. CNVs were called using fixed thresholds, representing the minimum log_2_ ratio for gains (0.40) and the maximum log_2_ ratio for losses (−0.55). An atypical log_2_-normalized coverage ratio <0.5 suggests a heterozygous deletion. 

#### 2.3.5. Sequence Data Processing: Fusion gene and IKZF1 microdeletion Analysis

Manta software (Illumina) was used to detect medium and large structural variants (>50 bp), including *IKZF1* deletions and translocations [28]. 

### 2.4. Analytical Validation: Sensitivity, Specificity, and Reproducibility Analysis

To determine the reproducibility of the custom NGS panel, three additional samples that did not belong to the series of patients described in the previous section were included in duplicate. In addition, two healthy control samples were added to avoid the detection of false positives.

Sensitivity and specificity were determined by comparing the variants reported by the custom NGS panel with those reported by the techniques routinely employed to detect each of the alterations. Mutational information obtained through other sequencing methodologies—454 Junior sequencing (Roche) and a targeted TruSeqCustom amplicon (TSCA) panel for ALL (pre-beta test plan for Illumina)—was used to validate the mutations. MLPA data were used to determine the sensitivity and specificity of the custom NGS panel for detecting CNVs, and karyotyping and FISH were used to validate the aneuploidies and fusion genes, respectively. To determine sensitivity, true positives (TPs) were taken into account, considering these to be the alterations detected by at least two techniques. False negatives (FNs) were considered to be those alterations reported by other techniques but not by NGS (sensitivity = TP/TP + FN). Specificity was calculated, taking into account the true negatives (TNs), considered to be alterations not reported by NGS or other techniques, and false positives (FPs), such as those alterations detected by NGS, which were not reported by any other technique (specificity = TN/TN + FP).

Data from 454 Junior sequencing included the mutational status of *JAK2* (E12-16, NM_004972.3), *TP53* (E4-11 NM_000546.5), *IL7R* (E6 NM_002185.5), *PAX5* (E2-3 NM_016734.3), *CRLF2* (E6, NM_022148.4), and *LEF1* (E2-3, NM_016269.5) genes. These data were previously published by Forero-Castro et al. [29]. ALL targeted TSCA panel included the mutational status of 52 genes (Montaño A. EHA-SWG, 2018) (Table S8). 

The PCR amplification-based method (amplicons) was used for the validation tests of the mutations. PCR libraries were prepared using a Nextera XT DNA sample preparation kit (Illumina) and indexed using a Nextera XT index kit (Illumina). The indexed libraries were then purified using Agencourt AMPure XP beads (Beckman Coulter, Brea, CA, USA), quality checked on a Bioanalyzer DNA 1000 chip (Agilent), and then quantified by fluorometry using a Qubit HS dsDNA assay kit (Invitrogen, Carlsbad, CA, USA). The libraries were then diluted to an equimolar concentration of 4 nM before pooling for sequencing. The pooled library concentration was confirmed for a final time by a fluorometric measurement before denaturing and sequencing. The pooled genomic libraries were then sequenced using the Illumina NextSeq or MiSeq platform (Illumina). Samples were processed using V3 MiSeq sequencing chemistry in a 2 × 151-bp run (8 samples per run).

Sanger sequencing (SS) in an ABI 3130 automated sequencer was used to validate polymorphism and fusion genes through the design of region-specific oligonucleotides. The specific forward and reverse primers were designed using Primer3 (http://bioinfo.ut.ee/primer3/). Genomic DNA was amplified with the Fast Start High Fidelity PCR System (Roche) following the manufacturer’s instructions, with some variations in the annealing temperature. DNA sequences were evaluated using Chromas Lite v2.1.1 (Technelysium, South Brisbane, Australia) and DS Gene v1.5 (Accerlys, San Diego, CA, USA) software. Data were analyzed using the annotations of genome version hg38/GRCh38. Table S9 shows the oligonucleotide pairs used for confirmation of fusion genes that were not previously reported by conventional techniques.

MLPA reactions were performed using the SALSA MLPA P335-B1 ALL-IKZF1 probe mix (MRC-Holland, Amsterdam, The Netherlands), according to the manufacturer’s instructions. DNA samples from three healthy donors were used as controls. The P335-B1 probe mix contains probes for the genes *IKZF1*, *CDKN2A/B*, *PAX5*, *EBF1*, *ETV6*, *BTG1*, and *RB1* and those from the X/Y PAR1 region. MLPA amplification products were analyzed on an ABI 3130xl Genetic Analyzer (Applied Biosystems, Foster City, CA, USA) with GeneMapper software V.3.7, using the Genescan 500LIZ internal size standard (Applied Biosystems). The copy number at each locus was estimated according to the method of Schwab et al. [30].

aCGH experiments were carried out in an aCGH 12X135K array platform (Roche NimbleGen, Madison, WI, USA). The segmentation analysis was performed using the CGHweb tool 8. The Database of Genomic Variants from Toronto (DGV, http://dgv.tcag.ca/dgv/app/home) was used to exclude DNA variations located in regions with defined CNVs. All copy number changes with more than 50% overlap with respect to those reported in the DGV were excluded. The data discussed in this publication have been deposited in NCBI’s Gene Expression Omnibus [24] and are accessible through GEO Series accession number GSE75671 (http://www.ncbi.nlm.nih.gov/geo/query/acc.cgi?token=ufczceaqvvudfup&acc=GSE75671).

## 3. Results

### 3.1. NGS Panel Performance

Results obtained from sequencing showed an average of 3.5 × 10^6^ reads/sample. At least 99.4% of the regions of interest were sequenced. The average sequence depths ranged from 800× to 20×. Due to the design, the regions corresponding to mutations had a median frequency of 342 (24.6–767.4) reads. The coverage depth obtained in the regions corresponding to mutation detection allowed the detection of variants in case of up to 5% VAF, thus allowing the presence of subclones to be determined. The regions with low VAF that obtained a depth coverage <250 were confirmed by amplicon-based sequencing. The regions corresponding to the SNPs associated with pharmacogenetics had a median read frequency of 73.7 (0–417.8). The CNVs and fusion gene regions had median read frequencies of 355.1 (81.9–647.1) and 570.2 (408.5–701.2), respectively.

A total of 868 variants (SNV/INDELS) were reported in the cohort of patients. Fourteen and 46 samples showed the presence of aneuploidy and CNVs in some of the genes studied, respectively. Thirty-five patients showed some of the recurrent fusion genes included in the panel design. No SNVs/INDELs were reported in control samples. The controls also showed an unaltered CNV profile. The NGS panel was highly reproducible compared with the duplicate sequenced samples and the results obtained from sequencing the different types of samples, such as fresh cells, cryopreserved cells, and tumor tissue (Table S10).

Validation assays determined sensitivity and specificity of 96.3% and 90.0% for SNV/INDEL detection compared with other sequencing methodologies; 89.7% and 100% for detecting fusion genes compared with FISH; 93.3% and 100% for aneuploidy detection compared with karyotyping, and 95.5% and 100% for the detection of CNVs compared with MLPA (Table S11).

Most of the alterations (>90%) detected with the custom NGS panel could be validated by other techniques (karyotyping, FISH, MLPA, aCGH, PCR, or other sequencing methodologies) and showed high levels of concordance. 

### 3.2. High Sensitivity and Reproducibility in SNV/INDEL Detection

**Figure 1:**
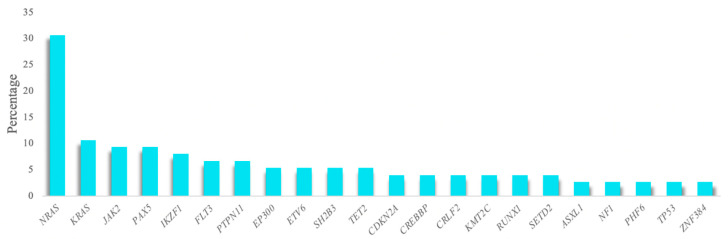
Genes most frequently mutated in the patient series. The histogram shows the genes detected in more than 2% of the patient series.

A total of 86.7% of the analyzed samples (65/75) featured a mutation in at least one of the genes studied, with a mean of 1.8 mutations per patient (range 0–6). One hundred and fifty-three variants were selected after applying the quality filters and removing SNPs and possible sequencing artifacts, of which most were non-synonymous SNVs (83.1%). These variants were detected in 55 of the genes studied. The most frequently altered gene was *NRAS*, which was detected in 30% of patients, followed by *KRAS*, *JAK2*, *PAX5,* and *IKZF1,* which were detected in more than 8% of the cohort (Figure 1). Over 70% of the mutations detected in the *NRAS* and *KRAS* genes were the hotspots located at codons 12 and 13. Furthermore, mutational hotspots R683 and T875 of *JAK2* were the most recurrent mutations observed in this gene (Table S12). 

The mutational status of *JAK2*, *TP53*, *IL7R*, *PAX5*, *CRLF2*, and *LEF1* genes, determined by 454 Junior sequencing in 62 of patients, and the mutational status of 52 genes, determined by an amplicon-based NGS panel (Illumina) in 18 patients, were used for custom NGS panel validation in mutation detection. In this way, the regions common to all panels were analyzed (454 Junior sequencing/amplicon panel vs. custom NGS panel). The analysis revealed 18 samples to have mutations in at least one of these common regions. The mutations reported by the NGS custom panel faithfully reproduced the alterations detected by the other sequencing methodologies, yielding an almost identical VAF in almost all samples (Table 1). 

In the ID24 sample, the variant p.E220D in the *CRLF2* gene was detected by all sequencing techniques but with discrepancies in the VAF. While similar VAFs (about 5%) were obtained with the custom panel and the amplicon panel, a higher VAF (75%) was observed with 454 Junior sequencing. Similarly, the mutation in *PAX5* (p.V267D) observed in the sample ID34 showed discrepancies in the VAF estimated by the different panels. On the other hand, a mutation in the *TP53* gene detected by the 454 Junior and amplicon panels was not detected by the custom NGS panel in ID25, probably because it was present in such a low percentage of cells (VAF ~3%). By contrast, two other mutations in the ID71 and ID72 samples were only detected by the custom NGS panel (Table 1). 

Mutations previously described in the REH cell line were also detected in the genes included in the panel: *TBLX1R1*, *IKZF1*, *NOTCH1*, *IL27*, *TP53*, *GATA3*, and *BCL11B*. These mutations were also maintained in several clones established from the REH cell line. Only two mutations of the parental cell line (REH) were not detected in the analyzed clones because they were established by single-cell sorting and were not present in 100% of the pool of parental cells (VAF ~30%) (Table S13).

SNPs associated with the response to drugs included in the panel were also successfully detected in genes, such as *MTHFR* and *SCOL1B1*, associated with methotrexate (MTX) metabolism, as well as in *NUDT15* and *ITPA*, to which 6-mercaptopurine (6-MP) toxicity levels are related. Table S14 shows the observed frequencies of pharmacogenetic SNPs in the patient cohort.

### 3.3. Accurate Aneuploidy Detection

**Figure 2:**
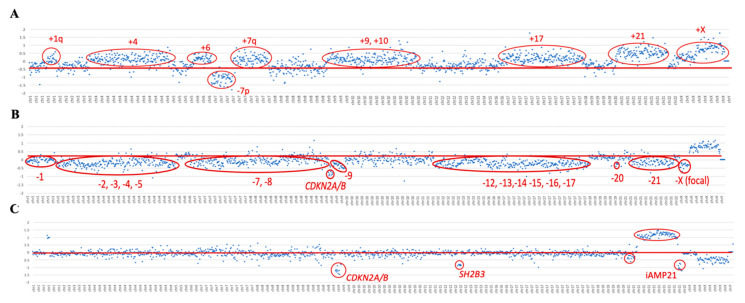
Dot plot of copy number variations (CNVs). This figure shows the dot plots of three samples for the detection of CNVs. The x-axis represents the chromosome regions included in the custom NGS panel, in increasing order from chromosome 1 to X. The y-axis shows the values of the log_2_ ratio. The red lines indicate the reference, and in the red circles, there are the chromosomal regions above or below the reference, showing the respective gain or loss of that region. (**A**) Dot plot of the ID37 sample, showing a high hyperdiploid clone with the gain of chromosomes 1q, 4, 6, 7q, 9, 10, 17, 21, and X, in addition to the loss of 7p. (**B**) Dot plot of the ID43 sample, showing a near-haploid clone with the loss of chromosomes 1, 2, 3, 4, 5, 7, 8, 12, 13, 14, 15, 16, 17, 20, and 21. (**C**) Dot plot of the ID44 sample, showing the loss of the *CDKN2A/B* and *SH2B3* genes, and of iAMP21.

To validate high hyperdiploid detection, 10 samples previously characterized by their karyotype and aCGH were included in the study. Five of them were selected because the presence of the high hyperdiploid clone was confirmed by aCGH, but not by karyotype, either because of a lack of mitoses or due to the failure of the malignant clone to grow. These 10 samples also included two samples in which the high hyperdiploid clone was detected in fewer than 20% of the cells per karyotype. Using the custom NGS panel, the high hyperdiploid clone was detected in all 10 samples analyzed, as reported by both the cytogenetic and aCGH approaches. In addition, two more samples with high hyperdiploid clone were identified, stratified within the *BCR/ABL1*-positive subgroup. Thus, the trisomies observed by NGS faithfully reproduced the findings observed by both techniques (Table 2). The NGS panel showed even greater sensitivity in detecting trisomies than aCGH, achieving more gains in all cases analyzed by the two methodologies. For high hyperdiploid detection, probes were distributed between the most frequently gained chromosomes in this subgroup of patients: chromosomes 4, 8, 10, and 21. However, due to the design of the panel, the probes distributed throughout the genome also enabled trisomies in other chromosomes to be detected (Figure 2A). The most frequently gained chromosomes were numbers 6 and 21: their trisomies were observed in 100% of patients, followed by those of chromosomes 10 and X, which were found in 92% of patients (Figure S1). 

The hypodiploid clones were studied with respect to the distribution of probes along chromosomes 7 and 17. Using the panel, the presence of a hypodiploid clone was detected in two of the three cases included in the study, which were confirmed by karyotyping. Within the subgroup of patients with hypodiploidy, a low hypodiploidy clone (31–39 chromosomes) was observed in the ID42 sample by karyotype and custom NGS panel. In the ID43 sample, more missing chromosomes were identified with the custom NGS panel than by the karyotype, it being possible to identify the presence of a near-haplotype clone (e.g., in Figure 2B). On the other hand, the karyotype of the ID41 sample showed the presence of a clone with low hypodiploidy, but this was not evident from the custom NGS panel. Interestingly, aCGH did not show any chromosomal losses in this sample either.

All three patients included in the study with iAMP21 reported by FISH were confirmed by NGS. The dot plot of these samples showed a characteristic profile in chromosome 21. In particular, a gain of the region described as the common region of amplification (CRA) was observed in all cases, in addition to other regions of chromosome 21 with losses (e.g., see Figure 2C).

**Figure 3:**
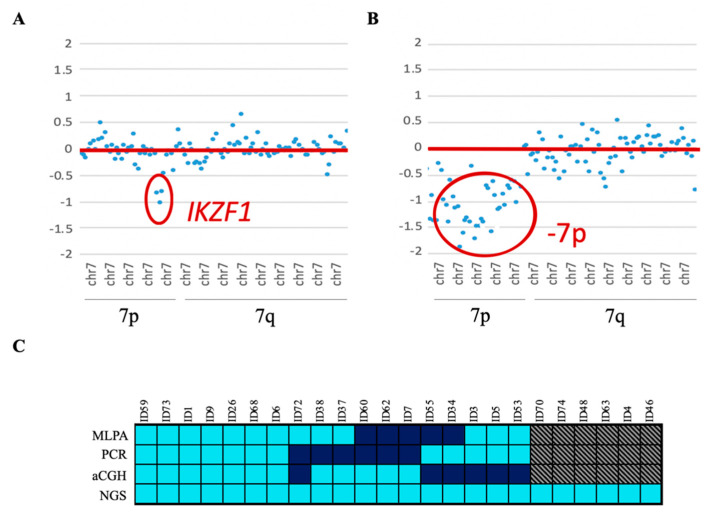
*IKZF1* loss. (**A**) Dot plot of CNV representation, showing *IKZF1* gene loss. (**B**) Dot plot of CNV representation, showing loss of 7p, which includes the *IKZF1* gene. The x-axis represents the chromosome regions included in the custom NGS panel, in increasing order from chromosome 1 to X. The y-axis shows the log_2_ ratios. The red lines indicate the reference, and the chromosomal regions above or below the reference are those inside the red circles, indicating the respective gain or loss of that region. (**C**) *IKZF1* loss detected by NGS compared with the results obtained by conventional multiplex ligation-dependent probe amplification (MLPA), PCR, and aCGH techniques. Turquoise, detected; dark blue, not detected; grey, no information available. Each column represents one patient.

The panel design also allowed the detection of CNVs in *IKZF1*, *CDKN2A/B*, *PAX5*, *BTG1*, *RB1*, and *ETV6* genes. These results were validated with the data available from MLPA and aCGH. Microdeletions of the *IKZF1* gene were also confirmed by PCR. Twenty-four patients showed *IKZF1*del, which was mainly enriched in the positive Philadelphia subgroup (33.3% of the cases). Eighteen of them were also reported by one of the other conventional techniques—MLPA, PCR, and/or aCGH. In addition, the NGS custom panel was able to detect all cases of *IKZF1* loss, which were discordant between the three techniques, without giving rise to any false-positive results. Four of these cases had a focal loss of the 7p arm that affected the entire *IKZF1* gene. These cases could not be detected by PCR as there was no region to which the designed primers could hybridize. The remaining six cases could not be confirmed because no previous genetic data were available (Figure 3).

One-third of the patients with the *IKZF1* deletion also featured a loss of the *CDKN2A/B* gene. This gene was lost in 20 patients, including all of those identified by the MLPA and/or aCGH (12/12). The loss of the *PAX5* gene was observed in 12 patients and was accompanied in a small majority of cases by the loss of *CDKN2A/B* (7/12), thus giving a detection rate of 92.3% for the cases confirmed by MLPA and/or aCGH. Only one case with *PAX5* loss reported by MLPA was not detected by NGS. This loss was also not reported by aCGH. On the other hand, 83.3% (9/11) of the cases with loss of *ETV6* confirmed by MLPA and/or aCGH were detected by NGS. These two patients had a focal loss of *ETV6* by aCGH, but this loss was also not detected by MLPA. In addition, the loss of *ETV6* was also detected in one patient without previous information. The loss of this gene was mainly detected in the *ETV6/RUNX1*-positive subgroup (7/10). The loss of the *BTG1* gene was detected in nine patients, thereby giving a detection rate of 85.7% of cases (6/7) that were confirmed by MLPA and/or aCGH. The sample in which the loss of *BTG1* by NGS was not detected had a focal loss by aCGH. The loss of *RB1* by NGS was confirmed in 100% of cases observed by MLPA and/or aCGH (2/2), in addition to two other cases with no previous data. Furthermore, the panel design allowed for the detection of CNVs in other genes included in the panel, such as *ERG*, *TP53*, and *VPREB1*, although these were not validated. No false positives were detected.

### 3.4. Fusion Gene Detection

The use of the custom NGS panel allowed the detection of the *ETV6/RUNX1* and *BCR/ABL1* fusion genes with high sensitivity and specificity. To study the various fusion genes, probes were added throughout the most frequently described regions where the breakpoints occur.

**Figure 4:**
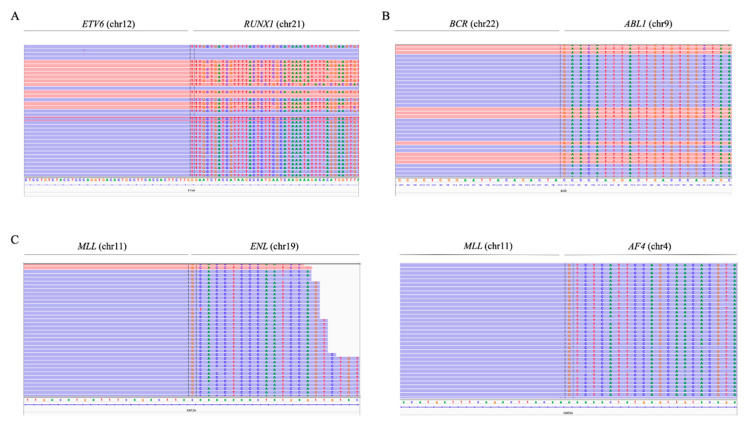
Fusion genes visualized with integrative genomics viewer (IGV). (**A**) *ETV6/RUNX1* fusion gene (sample ID52). In the left region, the reads were aligned with intron 5 of *ETV6*, while in the right, the reads were aligned with intron 3 of *RUNX1*. (**B**) *BCR/ABL1* fusion gene (sample ID63). In the left region, reads were aligned with intron 1 of *BCR*; to the right, reads were aligned with intron 1 of *ABL1*. (**C**) *MLL/ENL* fusion gene (sample ID30) and *MLL/AF4* fusion (sample ID27). In the left region, reads were aligned with intron 10 of *MLL*; to the right, reads were aligned with the intergenic region next to *ENL* and intron 3 of *AF4*, respectively. The position of the breakpoint was determined by MANTA software.

In the case of t(12;21)(p13;q22), probes were added along the intron 5 of *ETV6* (NM_001987.5). The presence of the fusion gene *ETV6/RUNX1* was detected in 11 samples, which represented 90% of the cases confirmed by FISH (9/10), and two other cases—ID52 and ID61—in which fusion was not reported (Figure 4A). PCR of the detected breakpoint confirmed the presence of the *ETV6/RUNX1* fusion gene in these samples (data not shown). In the sample ID13, in which the *ETV6/RUNX1* fusion gene reported by FISH was not detected, a different translocation was observed between chromosomes 5 and 12, involving the *ETV6* gene (Figure S2). The fusion gene *ETV6/RUNX1* was also analyzed in the REH cell line and the *ETV6/RUNX1* knockout (KO) clones established in previous studies [25]. The *ETV6/RUNX1* fusion gene was detected in 100% of cells from the REH cell line, as expected, and the truncator mutation was also found in 100% of the cells in the KO clones (data not shown).

In all ten samples in which the presence of the *BCR/ABL1* fusion gene was reported by cytogenetic techniques, the gene was also detected using the NGS panel. In addition, the fusion gene was detected by cytogenetics in a previously unreported sample (ID63) (Figure 4B). The presence of this fusion gene was confirmed by PCR (data not shown). For its detection, probes were distributed along intron 1 and exons 12-16 of *BCR* (NM_004327.4), which made it possible to distinguish between the two frequent types of rearrangements—the minor p190 (m-BCR) that occurs in *BCR* intron 1 and the major p210 (M-BCR) between *BCR* introns 12-16. The 90% (10/11) of patients showed m-BCR, while the other 10% (1/11) of the samples exhibited M-BCR.

To detect *MLL* rearrangements, probes along exons 9 to 12 of *MLL* (NM_005933.4) were included. Seventy percent of the cases confirmed by karyotype/FISH were also detected by NGS (7/10). The use of the panel also made it possible to determine the partner gene of *MLL* in these cases (Figure 4C). Five patients with *MLL*r detected by the custom NGS panel showed rearrangement with the *AF4* gene (chromosome 4), while in one patient, the rearrangement was observed with the *ENL* gene (chromosome 19) and, in another, with the *EPS15* gene (chromosome 1). By contrast, three cases with an *MLL* rearrangement confirmed by karyotype and/or FISH were not detected by the NGS panel.

To determine the detection sensitivity of the various fusion genes, samples were included in the study in which the fusion gene reported by FISH was found in a low percentage of cells. In this way, using NGS, it was possible to identify the fusion gene *ETV6/RUNX1*, *BCR/ABL1,* and *MLL* rearrangements in samples with percentages less than 12, 26, and 18%, respectively.

**Figure 5:**
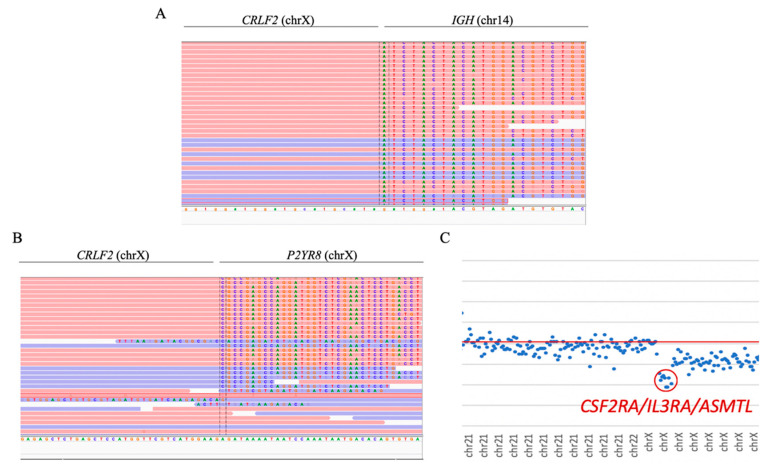
***CRLF2* rearrangement.** (**A**) *CRLF2/IGH* fusion gene visualized with IGV software (sample ID47). In the left region, reads were aligned with the intergenic region next to the *CRLF2* gene; to the right, reads were aligned with the 14q32.33 region. (**B**) *CRLF2/P2YR8* fusion gene visualized with IGV (sample ID48). In the left region, reads were aligned with the intergenic region next to the *CRLF2* gene; to the right, reads were aligned with intron 1 of *P2YR8* (NM_178129.5). The position of the breakpoint was determined by MANTA software. (**C**) Dot plot of sample ID70, showing the loss of the genes *CSF2RA*, *IL3R*, and *ASMTL*, resulting in the *CRLF2/P2RY8* fusion gene.

Both cases with *CRLF2* rearrangements were successfully detected. In sample ID47, the *CRLF2/IGH* fusion gene was observed as a product of t(14;X) (Figure 5A). Moreover, sample ID48 showed the presence of the *CRLF2/P2YR8* and *CRLF2/IGH* fusion genes. The presence of the *CRLF2/P2YR8* fusion gene was also determined by the detection of the loss of the genes *CSF2RA*, *IL3R*, and *ASMTL* (Figure 5B,C). *CRLF2* rearrangements were observed in four other samples in which the rearrangements were not reported by conventional techniques.

### 3.5. The Integrative Use of the Custom NGS Panel with Standard-of-Care Diagnostics Allowed the Stratification of More Patients

**Figure 6:**
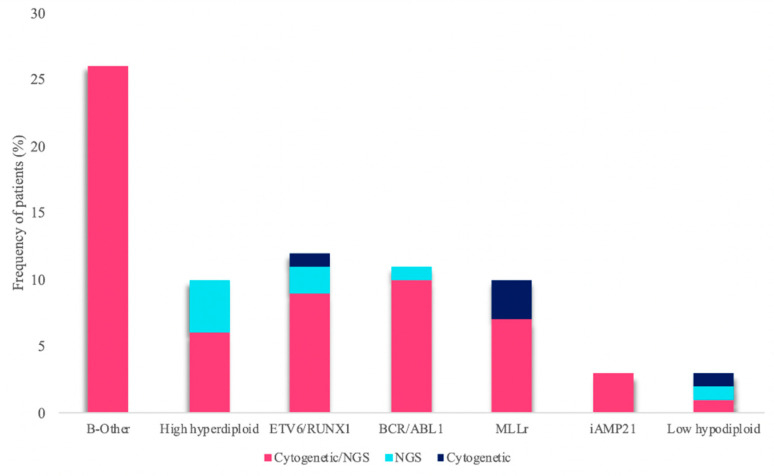
Frequency of genetic subtypes. The bar graph shows the percentage of genetic subtypes identified in the cohort of patients. Bars may be subdivided into three parts: pink, percentage of patients stratified through the combined use of cytogenetic techniques and NGS; turquoise, percentage of patients stratified exclusively by NGS; dark blue, stratified through the use of cytogenetic techniques only.

The integration of the use of the custom panel together with the conventional techniques routinely used in clinical practice was more successful at stratifying patients. In 28% (21/75) of the samples, the karyotype could not be analyzed correctly because there were insufficient metaphases or because the malignant clone did not grow. Some of the recurrent alterations were detected in three of the 29 B-other patients and were re-stratified into their corresponding groups. Of these alterations, 81.3% were detected by both methodologies, while 12% were detected only by the custom NGS panel, and 6.7% only by cytogenetic techniques (Figure 6). In addition, some secondary alterations were identified in 61.3% (46/75) of the samples by the use of the custom NGS panel.

## 4. Discussion

We presented the results of a study of the validation of a custom NGS panel designed to improve the stratification of B-ALL patients. Seventy-five samples from B-ALL patients, included in the study at the time of diagnosis, were genetically characterized by conventional techniques. The use of the custom NGS panel was able to detect the main genetic alterations associated with risk in B-ALL patients with high sensitivity and reproducibility, even in some cases that could not be detected by standard-of-care diagnostics. Secondary alterations were also detected by NGS, some of which were associated with prognostic risk, which could help refine the stratification of patients.

The integration of arrays and NGS techniques is currently the only option available for detecting the greatest possible number of alterations [18]. Nonetheless, these techniques are not available in all centers, above all, in less-developed countries, such as those in Latin America, where the survival rate remains very poor, even in children, mainly due to the lack of good clinical diagnosis [31]. In addition, these analyses usually involve the study of the entire genome, which makes it very difficult to interpret the data. Therefore, a tool that allows the detection of a large number of alterations in a single experiment and in a quick and inexpensive way could be very useful in clinical practice to help stratify patients and predict their response to treatments [32,33,34].

The NGS panel proved to be a robust tool for SNV/INDEL detection. The regions of the panel intended for detecting them had a high read coverage (mean coverage >300 reads), which allowed mutations to be identified in low percentages of cells. SNPs included in the panel design associated with drug responses were also successfully detected. Validation through data obtained by other sequencing techniques and ALL cell lines further demonstrated that the custom NGS panel is capable of detecting genetic variants with high accuracy and reproducibility.

On the other hand, the use of the NGS custom panel was successful at detecting cases with aneuploidies and CNVs. In the case of patients with a high hyperdiploid clone, the panel could detect even those cases that were not reported by karyotyping, and that required the use of aCGH to stratify them correctly. In addition, NGS was more sensitive at detecting trisomies than was aCGH. In the cases with hypodiploidy, the NGS panel also helped refine the stratification, making it possible to detect a case with near-haploid, who was diagnosed as low hypodiploidy by karyotype. These cases sometimes pose a dilemma during the diagnostic process because a near-haploid or low hypodiploid clone can ‘double-up’ and appear as hyperdiploid/near-triploid when karyotyped [15]. In addition, iAMP21 was detected in all the cases studied, making it possible to distinguish it from cases with trisomy 21 based on their characteristic profile in the representation of the dot plot of the CNV. In this way, the findings observed by NGS faithfully reproduced what was observed by karyotyping and/or aCGH, but with even greater sensitivity than with the combination of the two. CNVs were also detected in key genes involved in the evolution of B-ALL, effectively reproducing the results observed by MLPA and/or aCGH. NGS also made it possible to detect more genes than those included in the commercial panels of MLPA, thereby avoiding the main limitation of this technique.

Finally, we evaluated the sensitivity and effectiveness of the custom NGS panel in detecting the main fusion genes associated with B-ALL patients: the *ETV6/RUNX1* fusion gene (reported in 25% of children and 1–3% of adults), the *BCR/ABL1* fusion gene (reported in 10% of children and 20% of adults), and *MLL* rearrangements (reported in 6% of children and 9% of adults) [1,2,35]. The NGS panel was able to identify all fusion genes included in the panel. Furthermore, it enabled fusion genes to be detected with high sensitivity, confirming the presence of the various fusion genes in patients with the fusion in a small percentage of cells, detecting even cases that were not reported by conventional techniques. In *MLL*r cases, the NGS panel only detected 70% of the cases confirmed by cytogenetics because only the region where the most frequent *MLL* breakpoints occur was included in the panel design. Twenty percent of *MLLr*-positive patients might present the breakpoint in a different position and, therefore, not be detected by the panel.

The custom NGS panel proved to be a powerful tool for the detection of the main genetic alterations present in B-ALL patients, on which the current classification systems of this disease are based. The use of NGS allowed further refinement of the prognosis of the already established biological groups, especially the high-risk subgroup. In the *BCR/ABL1*-positive cases, we were able to distinguish between the different transcripts because it was possible to identify the *BCR* breakpoint. Although no studies have demonstrated the clinical and prognostic significance of the different *BCR/ABL1* transcripts in B-ALL, it has been demonstrated in CML patients [36]. On the other hand, by using the panel, it was possible to determine the *MLL* partner in rearrangement cases, which is known to have a clinical impact [37,38].

The correct stratification of patients into different risk groups is of vital importance because it has a direct impact on therapeutic decision-making and because poor classification can lead to the choice of inappropriate treatment or dose. The integration of this NGS panel with standard-of-care diagnostics stratified more patients than was possible by using them separately. Therefore, the implementation of this NGS panel in clinical practice could help improve patient stratification, overcoming the limitations of the conventional techniques commonly used in clinical diagnosis. Detection of secondary alterations, such as somatic mutations, SNPs associated with pharmacogenetics, CNVs, and new fusion genes, could also help improve stratification.

The panel design includes the coding regions of up to 150 genes involved in the evolution of B-ALL. Although only *TP53* mutations are currently taken into account in clinical practice, the mutational status of key genes could also be considered in the near future. These genes often affect key pathways in the pathogenesis of the disease, such as the *RAS*, *JAK/STAT*, and *PI3K* pathways, which are known to be promising therapeutic targets [18,39,40,41]. A total of 164 SNPs associated with pharmacogenetics were also included, which could provide valuable information for clinicians when making therapeutic decisions.

Some CNVs have already been established as new prognostic markers. In particular, the loss of the *IKZF1* gene has been associated with an adverse prognosis and with an even worse prognosis when accompanied by other alterations, such as *CDKN2A/B* deletion, also known as “*IKZF1*^plus^” [42,43]. The *IKZF1* deletion has also been reported recurrently in Ph-like patients, which is frequently accompanied by the loss of other genes, such as *PAX5* and *BTG1*, as well as somatic mutations, all of which are included in the NGS panel [44]. *CRLF2* rearrangements have frequently been reported in this subgroup of patients, which has also been associated with a worse prognosis [45,46,47]. The detection of all these alterations could, therefore, be very useful for identifying the genetic signature of Ph-like patients, for whom there is not yet a well-established or standardized diagnostic method. Although transcriptomic analysis is currently the gold standard for the diagnosis of these patients, it remains a challenge in the clinical routine of many diagnostic centers [48]. Many of these alterations are also possible therapeutic targets, which is of great interest, especially when dealing with patients who have a very poor prognosis.

**Figure 7:**
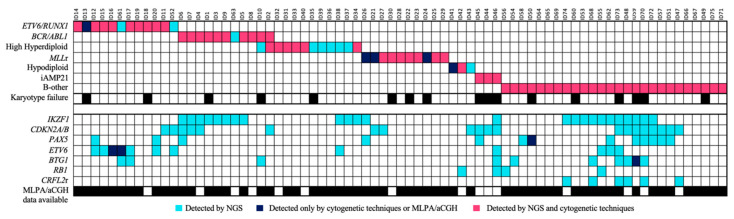
**Genetic findings from the combined analysis of the custom NGS panel and standard-of-care diagnostics.** The figure summarizes the genetic alterations observed in the series of B-ALL patients, where columns and rows represent patients and genetic alterations, respectively. The upper box shows the alterations that allow patients to be classified by biological risk groups. Turquoise, dark-blue, and pink boxes, respectively, indicate the alterations detected exclusively by the custom NGS panel and cytogenetic techniques and those detected by both. In the lower box, the different secondary alterations detected correspond to the loss of the genes indicated in the different rows and to the rearrangements of the *CRFLF2* gene.

In conclusion, the design of this custom panel allowed the detection of the main genetic alterations present in B-ALL patients, with which they could be stratified into different risk groups. In addition, a series of secondary alterations of high prognostic value, many of which are also promising therapeutic targets, were identified. The use of this NGS panel could facilitate better patient stratification and the refinement of the established biological groups. Our results demonstrated the advantages of NGS for managing patients, improving detection sensitivity, and solving the problems of conventional techniques (Figure 7). The integration of the NGS panel in clinical practice could complement the standard diagnostic techniques (karyotyping and FISH), while avoiding the use of additional techniques, thereby reducing costs and response time. In addition, the identification of cooperating alterations could be useful in future research that aims to detect new biomarkers and predictors of response to treatment.

## Figures and Tables

**Table 1:** Correlation between mutations detected by the custom NGS panel and 454 Junior sequencing. The table lists the patients who presented some mutations in the genes studied by sequencing with 454 Junior. The first column contains the patient identifier (ID) in bold, followed by the gene, altered nucleotide in coding sequence (CDS mutation), an altered amino acid (AA mutation), and variant allele frequency (VAF) detected by 454 Junior sequencing and the custom NGS panel.

ID	Gene	CDS Mutation	AA Mutation	VAF (%)		
				454 Junior (Roche)	TSCA (Ilumina)	Custom NGS Panel
**ID5**	***RUNX1***	**c.G239A**	p.R80H	-	30.58	40.76
**ID43**	*TP53*	c.560-1G->A	Splice_Intron 5 SA	41	-	42.27
**ID23**	*FLT3*	c.G2503T	p.D835Y	-	19.24	12.86
	*KRAS*	c.G35T	p.G12V	-	19.29	14.68
	*JAK3*	c.C23T	p.T8M	-	55.23	41.18
**ID64**	FLT3	c.G580A	p.V194M	-	49.33	43.79
**ID73**	*CRLF2*	c.695T>G	p.F232C	21.5	-	25.69
	*JAK2*	c.2049A>T	p.R683S	13.79	-	14.96
**ID24**	*CRLF2*	c.660G>T	p.E220D	75.73	5.28	5.19
**ID25**	*PAX5*	c.399T>A	p.S133R	20.4	31.22	23.08
	*TP53*	c.818G>C	p.R273P	3.5	2.72	0
**ID41**	*PAX5*	c.215A>G	p.Y72C	54.65	53.98	50.66
	*PAX5*	c.239C>G	p.P80R	39.62	0	44.13
**ID60**	*JAK2*	c.2047A>G	p.R683G	11.79	5.51	6.67
	*JAK2*	c.2624C>A	p.T875N	-	16.68	16.85
**ID74**	*JAK2*	c.2047A>G	p.R683G	40	-	41.10
**ID40**	*ETV6*	c.1165_1166del	p.M389fs	-	40.41	35.19
	*FLT3*	c.T1733C	p.M578T	-	45.13	36.51
**ID42**	*TP53*	c.G242A	p.R81Q	-	82.16	88.82
	*NF1*	c.C4537T	p.R1513X	-	82.17	69.35
	*CRLF2*	c.G394A	p.V132M	-	46.61	46.18
**ID26**	*SH2B3*	c.C464T	p.P155L	-	52.52	51.58
**ID72**	*NRAS*	c.G35A	p.G12D	-	0	3.97
**ID71**	*PHF6*	c.G55A	p.R129X	-	29.64	18.42
	*RUNX1*	c.C385T	p.A19T	-	0	46.68
**ID34**	*PAX5*	c.T800A	p.V267D	-	84	48.12
	*KRAS*	c.G35T	p.G12V	-	14.4	13.39
**ID20**	*NRAS*	c.G35A	p.G12D	-	8.48	4.49
**ID29**	*NRAS*	c.G35A	p.G12D	-	6.41	5.13

**Table 2:** High hyperdiploid cases detected by the custom NGS panel. Twelve cases (listed by ID in bold) with high hyperdiploid clone detected by next-generation sequencing (NGS), with their corresponding karyotype and fluorescent in situ hybridization (FISH), microarray-based comparative genomic hybridization (aCGH), and custom NGS panel results are shown.

ID	Karyotype	aCGH	NGS
**ID2**	55-60,XY,+Y,+4,+5,+7,+8,t(9;22)(q34;q11),+9,+11,add(12)(p12) [15]	+4, +18, +X	+4, +6, +10, +11, +14, +17, +21, +X
**ID10**	Karyotype failure	+2, +4, +6, +10, +14, +21	+2, +4, +6, +10, +14, +21, +X
**ID31**	51-58,XX,+5,+6,+9,+10,+21 [14]/46,XX [6]	ND	+4, +6, +14, +17, +21, +X
**ID32**	48-52,XX,+2,+6,+8,+10,+12,+21 [12]/46,XX [3]	ND	+4, +6, +8, +9, +10, +17, +21, +X
**ID33**	51,XX,+21,4mar [4]/46,XX [17]	+10, +18, +21	+5, +6, +9, +10, +17, +21, +X
**ID34**	50-52,XX,+10,+17,+18,+21,+mar [3]/46,XX [8]	+10, +14, +21	+6, +8, +9, +10, +14, +19, +21, +X
**ID35**	Karyotype failure	+4, +5, +6, +8, +10, +14, +21, +X	+4, +5, +6, +8, +10, +14, +17, +21, +X
**ID36**	46,XX [10]	+6, +8, +13, +14, +19, +21	+5, +6, +8, +10, +12, +13, +14, +19, +21
**ID37**	46,XX [10]	+4, +6, +9, +10, +14, +17, +21	+4, +6, +9, +10, +17, +21, +X
**ID38**	46,XX [6]/46,XX,add(3)(q)(21) [2]	+10, +14, +17, +21	+4, +6, +9, +10, +17, +21, +X
**ID39**	46,XY [10]	+14, +17, +18, +21, +22, +X	+4, +6, +9, +10, +14, +17, +21, +22, +X
**ID40**	60,XY,+X,+4,+5,+6,+8,+10,+10,+16,+17,+18,+21,+22,+mar [14]	ND	+4, +5, +6, +8, +10, +16, +17, +21, +22, +X

## Supplementary Materials

Additional information is available online at https://www.mdpi.com/2075-4426/10/3/137/s1. Table S1: Genetic characteristics of the patients; Table S2: Clinical and demographic characteristics of the patient cohort; Table S3: List of genes included in the panel design for mutation analysis; Table S4: Chromosomal regions for detecting gene fusions; Table S5: Chromosomes mapped to aneuploidy detection; Table S6: Genes for CNV detection; Table S7: Pharmacogenomic SNPs included; Table S8: Genes included in the targeted TruSeqCustom amplicon (TSCA) panel for ALL (pre-beta test plan for Illumina); Table S9: Oligonucleotide pair for detecting gene fusions; Table S10: List of variants detected in duplicate-sequenced samples; Table S11: Sensitivity and specificity of detection of different types of alterations; Table S12: List of detected mutations; Table S13: Mutations described in REH cell line; Table S14: Frequency of pharmacogenetic SNPs detected in the patient cohort; Figure S1: Frequency of trisomies in high hyperdiploidy cases; Figure S2: t(5;12)(q35;p13) visualized with IGV.
