# Supplementary material for: Comprehensive Custom NGS Panel Validation for the Improvement of the Stratification of B-Acute Lymphoblastic Leukemia Patients
Source: J Pers Med. 2020 Sep 21;10(3):137. doi: 10.3390/jpm10030137 (PMC7565730; doi:10.3390/jpm10030137)
Supplement: Supplementary file 1 [file jpm-10-00137-s001.pdf]

Supplementary information

Comprehensive Custom NGS Panel Validation for the Improvement of the Stratification of B-Acute Lymphoblastic Leukemia Patients

Adrián Montaño [1], Jesús Hernández-Sánchez [1], Maribel Forero-Castro [2], María Matorra-Miguel [1], Eva Lumbreras [1], Cristina Miguel [1], Sandra Santos [1], Valentina Ramírez-Maldonado [1], José Luís Fuster [3], Natalia de Las Heras [4], Alfonso García-de Coca [5], Magdalena Sierra [6], Julio Dávila [7], Ignacio de la Fuente [8], Carmen Olivier [9], Juan Olazabal [10], Joaquín Martínez [11], Nerea Vega-García [12], Teresa González [7], Jesús María Hernández-Rivas [1,7,*] and Rocío Benito [1,*]

[1] IBSAL, IBMCC, Universidad de Salamanca, CSIC, Centro de Investigación del Cáncer (CIC), Salamanca, 37007, Spain; adrianmo18@gmail.com (A.M.); jesus807@gmail.com (J.H.-S.); mariammzamora@gmail.com (M.M.-M.); a21093@usal.es (E.L.); cristinamiguelgarcia@gmail.com (C.M.); sandruskism90@gmail.com (S.S.); vramirem@gmail.com (V.R.);

[2] Escuela de Ciencias Biológicas (Grupo de investigación GICBUPTC), Universidad Pedagógica y Tecnológica de Colombia, Tunja, 150003, Colombia; maribel.forero@uptc.edu.co

[3] Sección de Oncohematología Pediátrica, Hospital Clínico Universitario Virgen de la Arrixaca; Instituto Murciano de Investigación Biosanitaria (IMIB), Murcia, 30120, Spain; josel.fuster@carm.es

[4] Departamento de Hematología—Hospital Virgen Blanca, León, 24008, Spain; nherasr@saludcastillayleon.es

[5] Departamento de Hematología—Hospital Clínico de Valladolid, Valladolid, 47003, Spain; agarciaco@saludcastillayleon.es

[6] Complejo Sanitario de Zamora, Zamora, 49022, Spain; msierrap@saludcastillayleon.es

[7] Departamento de Hematología—Hospital Universitario de Salamanca, Salamanca, 37007, Spain; juldaval@hotmail.com, teresa.gonzalez.mart@gmail.com

[8] Departamento de Hematología—Hospital Rio Hortega, Valladolid, 47012, Spain; ifuentegr@saludcastillayleon.es

[9] Servicio de Hematología y Hemoterapia—Complejo Sanitario de Segovia, Segovia, 40002, Spain; colivierco@gmail.com

[10] Departamento de Hematología—Hospital Universitario de Burgos, Burgos, 09006, Spain; jolazabal@saludcastillayleon.es

[11] Departamento de Hematología—Hospital Universitario 12 de Octubre, Madrid, 28041, Spain; jmarti01@med.ucm.es

[12] Laboratorio de Hematología, Instituto de Investigación, Hospital Sant Joan de Déu, Barcelona, 08950, Spain; nvega@fsjd.org

* Correspondence: jmhr@usal.es (J.M.H.-R.); beniroc@usal.es (R.B.)

Table S1. Genetic characteristics of the patients. This table shows the genetic characteristics of the B-ALL patients included in the study. In the first column the genetic subtype, followed by the patient identifier, the karyotype, the FISH, the findings observed by MLPA and changes greater than 5Mb detected by aCGH. Not done or Not data (ND), Male (M), Female (F).

Genetic subtype	ID	Sex/ Age (years)	Karyotype	FISH	MLPA	aCGH >5Mb
BCR/ABL1	ID1	M/70	Karyotype failure	LSI BCR/ABL1 fusion (54%)	*IKZF1*	+19
	ID2	M/76	55-60,XY,+Y,+4,+5,+7,+8,t(9;22)(q34;q11),+9,+11,add(12)(p12)[15]	LSI BCR/ABL1 Clonal - 9q34/ABL1 gain (89%), BCR/ABL1 fusion (89%), LSI MLL-r clonal - 11q23/MLL gain (28%)	ND	+4, +18, +X, -19
	ID3	M/34	46,XY,t(9;22)(q34;q11)[7]/46,XY[5]	ND	*IKZF1*	+17, +X
	ID4	M/50	46,XY,t(9;22)(q14;q11)[10]/46,XY[5]	LSI BCR/ABL1 fusion (97%)	ND	ND
	ID5	M/47	46,XY,t(9;22)(q34;q11)[7]/47,XY,+8,t(9;22)(q34;q11)[2]/46,XY[5]	LSI BCR/ABL1 fusion (65%)	Normal	+8
	ID6	M/ND	46,XY[6]	LSI BCR/ABL1 fusion (82.5%)	*IKZF1, CDKN2A, PAX5*	-9
	ID7	M/70	46,XY,t(9;22)(q34;q11)[8]/46,XY[2]	LSI BCR/ABL1 fusion (87%)	*CDKN2A*	Normal
	ID8	F/72	46,XX,t(9;22)(q34;q11)[2]/46,XX[10]	LSI BCR/ABL1 fusion (26%)	ND	ND
	ID9	M/70	46,XY,t(9;22)(q34;q11)[13]/46,XY[8]	LSI BCR/ABL1 fusion (93%)	*IKZF1*	Normal
	ID10	F/45	Karyotype failure	LSI BCR/ABL1 fusion (98%)	*BTG1*	+2, +4, +6, +10, +14, +21, -12p
ETV6/RUNX1	ID11	M/14	46,XY[15]	ETV6/RUNX1 fusion (80%), LSI 12p13/ETV6 gain (33%), 21q22/RUNX1 gain (76%), LSI 11q23/MLL gain (26%), LSI 9q34/ABL1 gain (48%), 22q11.2/BCR gain (60%)	*CDKN2A, PARP1*	+21

	ID12	F/3	46,XX[12]	ETV6/RUNX1 fusion (12%), LSI - 21q22/RUNX1 gain (63%)	*PAX5, ETV6*	+21
	ID13	F/5	Karyotype failure	LSI ETV6/RUNX1 fusion (81%)	*PARP1*	-X
	ID14	M/3	46,XY[10]	LSI ETV6/RUNX1 fusion (95,5%)	Normal	Normal
	ID15	M/2	48,XY,+11,+17[3]/44,XY,-13,-15[2]/46,XY[14]	Normal. ETV6/RUNX1 fusion positive by RT-PCR	*ETV6*	+X, -12p
	ID16	M/2	46,XY[10]	LSI ETV6/RUNX1 fusion (94%)	Normal	Normal
	ID17	F/5	46,XX[10]	LSI ETV6/RUNX1 fusion (96%)	*BTG1, ETV6*	+19
	ID18	M/2	Karyotype failure	LSI ETV6/RUNX1 fusion (90%)	ND	ND
	ID19	F/15	47,XX,+21c[15]/46,XX[3]	LSI ETV6/RUNX1 fusion (88,5%), LSI 21q22/RUNX1 gain (100%)	Normal	Normal
	ID20	F/14	Karyotype failure	LSI ETV6/RUNX1 fusion (96%)	*PAX5, ETV6*	+21, -6q
***MLL*r**	ID21	F/47	46,XX,t(4;11)(q21;q23)[14]	Normal	Normal	Normal
	ID22	M/46	Karyotype failure	LSI MLL-r (95,5%)	Normal	Normal
	ID23	F/0	46,XX,inv(11)(q12q23),del(12)(p13)[7]/46,XX[13]	LSI MLL-r (93%)	Normal	Normal
	ID24	M/60	Karyotype failure	LSI MLL-r (73%)	*PARP1*	+6
	ID25	F/31	46,XX,t(4;11)(q21;q23)[10]	Normal	Normal	Normal
	ID26	M/16	46,XY,t(4;11)(q21;q23)[7]/46,XY[3]	LSI MLL-r (83%)	*IKZF1, PAX5*	-17, -19, -22
	ID27	M/55	46,XY,t(4;11)(q21;q23)[3]/46,XY[10]	LSI MLL-r (75%)	*CDKN2A*	Normal
	ID28	F/0	47,XX,t(4;11)(q21;q23),+mar[23]	LSI MLL-r (83%)	Normal	Normal
	ID29	F/0	40-42,XX[6]/46,XX[6]	LSI MLL-r (18%)	Normal	Normal
	ID30	M/0	ND	LSI MLL-r (65%)	ND	ND
	ID31	F/5	51-58,XX,+5,+6,+9,+10,+21[14]/46,XX[6]	LSI 21q22/RUNX1 gain (89%)	Normal	+4, +6, +14, +17q, +18, +21, +X, -17p

**High hyperdiploid**	ID32	F/4	48-52,XX,+2,+6,+8,+10,+12,+21 [12]/46,XX[3]	ND	ND	ND
	ID33	F/3	51,XX,+21,4mar[4]/46,XX[17]	LSI 21q22/RUNX1 gain (94%), LSI 9q34/ABL1 gain (51%), LSI 11q23/MLL gain (25,5%)	Normal	+10, +18, +21
	ID34	F/4	50-52,XX,+10,+17,+18,+21,+mar[3]/46,XX[8]	LSI 21q22/RUNX1 gain (48.5%)	*IKZF1*	+10, +14, +21
	ID35	F/2	Karyotype failure	LSI 12p13.2/ETV6 gain (16%), LSI 21q22.12/RUNX1 gain (55%)	Normal	+4, +5, +6, +8, +10, +14, +21, +X, -19, -20, -22
	ID36	F/47	46,XX[10]	LSI 2p13.2/ETV6 gain (90%), LSI 21q22.12/RUNX1 gain (90%), LSI MYC gain (62%), LSI IGH gain (60%)	Normal	+6, +8, +13, +14, +19, +21, -17q, -X
	ID37	F/4	46,XX[10]	Normal	*IKZF1*	+1q, +4, +6, +7q, +9, +10, +14, +17, +21, -7p
	ID38	F/6	46,XX [6]/46,XX,add(3)(q)(21)[2]	ND	*IKZF1, ETV6*	+10, +14, +17, +21
	ID39	M/16	46,XY [10]	Normal	Normal	+10q, +14, +17, +18, +21, +22, +X
	ID40	F/4	60,XY,+X,+4,+5,+6,+8,+10,+10,+16,+17,+18,+21,.MOB+22,+mar[14]	ND	ND	ND
**Hypodiploid**	ID41	M/34	39-42,XY[cp12]	Normal	Normal	Normal
	ID42	ND	39,XX,-3,-7,-13,-15,-16,-17,-20[18]/46,XX[2]	Normal	ND	ND
	ID43	F/71	33-40,XX,add(4)(q33)[9]/46,XX[1]	ND	*CDKN2A*	Normal
**iAMP21**	ID44	M/13	Karyotype failure	LSI 21q22/RUNX1 gain (98%)	ND	ND
	ID45	F/8	Karyotype failure	LSI 21q22/RUNX1 gain (90%)	ND	ND
	ID46	F/11	Karyotype failure	LSI 21q22/RUNX1 gain (55%)	ND	ND
**B-Other**	ID47	ND	47,XX,t(X;14)(p22;q32),+der(X)t(X;14),inc[6]/47,idem,i(17)(q10)[1]	LSI CRFL2/IGH fusion (90%)	ND	ND
	ID48	F/58	46,XX[20]	LSI CRFL2/IGH fusion (87%)	ND	ND
	ID49	M/3	Karyotype failure	LSI 21q22/RUNX1 gain (82%)	Normal	X+

ID50	F/6	Karyotype failure	Normal	Normal	Normal
ID51	F/39	46,XX[22]	Normal	*CDKN2A*	+1q, +10p, +19p, -9p, -17p
ID52	F/7	46,XX[4]/46,XX,del(6)(q15q23) [6]	ND	*CDKN2A, ETV6*	-9p
ID53	M/16	46,XX[12]	Normal	*IKZF1*	+14
ID54	F/10	46,XX[8]	Normal	*BTG1*	Normal
ID55	F/6	46,XX[22]	Normal	*ETV6*	-13
ID56	M/4	46,XY[20]	Normal	*RB1*	Normal
ID57	M/7	46,XY[22]	Normal	*CDKN2A, PAX5*	-9p
ID58	F/1	46,XX[14]	Normal	*PAX5*	+1q
ID59	M/4	Karyotype failure	Normal	*IKZF1, CDKN2A, PAX5*	-9p, -20q
ID60	M/61	46,XY[13]	Normal	*IKZF1*	ND
ID61	M/5	46,XY,add(12)(q22)[16]/46,XY[4]	ND	*BTG1*	Normal
ID62	F/1	46,XX[5]/46,XX[15]	Normal	*PAX5*	-7p
ID63	M/8	46,XY[11]	ND	ND	ND
ID64	F/2	46,XX[10]	ND	Normal	-X
ID65	F/15	46,XX[2]/46,XX,t(3;17)(q13;p13)[10]	ND	Normal	-X
ID66	F/6	46,XX[10]	Normal	Normal	Normal
ID67	M/1	47,XY,+21[3]/46,XY[18]	Normal	Normal	Normal
ID68	M/25	46,XY [17]/46,XY,del(14)(q12)[8]	Normal	*IKZF1, BTG1*	-8p
ID69	F/5	46,XY[8]	ND	Normal	Normal
ID70	M/5	Karyotype failure	Normal	ND	ND
ID71	F/ND	Karyotype failure	Normal	*CDKN2A*	ND
ID72	M/1	46,XY[12]	ND	*IKZF1, JAK2, CDKN2A, PAX5*	-9p, -X

	ID73	M/20	Karyotype failure	Normal	*IKZF1, CDKN2A, BTG1, ETV6*	+X
	ID74	M/3	47,XY,+21c	ND	ND	ND
	ID75	F/4	49,XX,+10,+19,+20[7]/46,XX[3]	Normal	Normal	Normal

Table S2. Clinical and demographic characteristics of the patient cohort.

Parameter	N	%	**Median** (range)
**Gender** (n=73)			
Male	35	47.9	
Female	38	52.1	
**Age (years)**			7 (0 - 76)
Age <18	50	66.7	
Age >18	25	33.3	
**Biochemical data**			
Hb (g/L)			84 (26 - 144)
Platelets (x 10^9/L)			55 (7 - 580)
WBC >30 (x10^9/L) (n=55)	28	50.1	
Blast in BM			85 (10 - 98.3)
MRD >0.01% (n=59)	27	45.8	

Table S3. List of genes included in panel design for mutation analysis.

ID gene	Exon	Transcript
ABL1	4 - 10	NM_005157.5
ABL2	Full CDS	NM_001136000
ADARB2	5 - 7	NM_018702.3
AFF3	14, 17	NM_002285.2
ASMTL	4, 7, 10 - 12	NM_004192.3
ASXL1	13	NM_015338.5
ATM	3	NM_000051.3
ATP10A	10	NM_024490.3
ATRX	8, 17, 22, 30	NM_138270.3
BCOR	4, 13	NM_017745.5
BIRC3	4	NM_001165.4
BLNK	17	NM_013314.3
BRAF	1, 3, 10, 11, 15, 17, 18	NM_004333.5
CBL	8, 9	NM_005188.3
CCT6B	4, 8	NM_006584.3
CDCP1	3	NM_022842.4
CDH17	16	NM_004063.3
CDKN2A	1, 2	NM_000077.4
CDX2	3	NM_001265.5
CENPE	24	NM_001813.2
CLCA4	6	NM_012128.3
CREBBP	1, 4, 6, 10, 14, 16 - 19, 21, 24 - 28, 30, 31	NM_004380.2
CRLF2	Full CDS	NM_022148
CSF3R	14	NM_000760.3
CTCF	6	NM_006565.3
DCK	2, 3	NM_000788.2
DIS3	Full CDS	NM_014953
DNAH2	71	NM_020877.3
DNM2	Full CDS	NM_004945

DNMT3A	13 - 23	NM_022552.4
DOT1L	5, 15	NM_032482.2
DRD3	2, 3	NM_000796.5
DTX1	6	NM_004416.2
EBF1	3, 13	NM_024007.4
ECT2	20	NM_018098.5
ECT2L	Full CDS	NM_001077706
EED	8, 12	NM_003797.4
EP300	2, 11, 12, 14, 30	NM_001429.3
EPOR	2, 3	NM_000121.3
ERG	11	NM_004449.4
ETV6	Full CDS	NM_001987
EZH2	1, 3, 5, 7, 13 - 19	NM_152998.2
FANCD2	6	NM_033084.4
FAT1	2, 8	NM_005245.3
FAT3	6, 23	NM_001008781.2
FBL	2	NM_001436.3
FBXW7	Full CDS	NM_033632
FLT3	5, 8, 9, 12 - 16, 19 - 21	NM_004119.2
FOXO4	1	NM_005938.3
GATA2	3	NM_032638.4
GATA3	5, 6	NM_002051.2
GATA4	6	NM_002052.4
GSTM1	Full CDS	NM_000561
GSTP1	5	NM_000852.3
HDAC2	6	NM_001527.3
HES1	3	NM_005524.3
HPRT1	7	NM_000194.2
HRAS	2	NM_005343.2
IDH1	4, 7	NM_005896.3
IDH2	4	NM_002168.3
IKZF3	Full CDS	NM_012481

IL7R	3, 5, 6	NM_002185.4
IRF8	7, 8	NM_002163.2
JAK1	9, 10, 12 - 23	NM_002227.3
JAK2	14, 16, 20, 21, 24	NM_004972.3
JAK3	2, 4, 5, 10 - 13, 16, 18, 19	NM_00215.3
KLHL6	6	NM_130446.3
KMD6A	15, 16, 24, 26, 28	NM_021140.3
KMT2C	7, 14, 18, 53	NM_170606.2
KMT2D	11, 28, 38, 43, 53	NM_003482.3
KRAS	1 - 4	NM_004985.4
LEF1	3, 4	NM_016269.4
LLGL1	14	NM_004140.3
MAPK1	4, 9	NM_002745.4
MDM4	3	NM_002393.4
MOV10L1	18	NM_018995.2
MPL	10, 12	NM_005373.2
MST1	14	NM_020998.3
MYBL2	6, 7	NM_002466.3
MYC	2	NM_002467.5
NANOG	3	NM_024865.3
NBN	6	NM_002485.4
NCOR1	5, 15	NM_006311.3
NF1	9, 10, 12, 18, 19, 21, 23, 25 - 29, 31, 33, 34, 36 - 38, 41, 42, 44, 49, 52	NM_00267.3
NOTCH1	7, 11, 17, 25 - 28, 33, 34	NM_017617.4
NOTCH2	11, 34	NM_024408.3
NOTCH3	33	NM_00435.2
NR3C1	2, 6 - 8	NM_000176.2
NR3C2	2	NM_000901.4
NRAS	1- 3	NM_002524.4
NSD2	4 - 20	NM_133335.3
NT5C2	4, 11, 15	NM_012229.4

NTRK3	3, 13	NM_002530.3
NUDT15	1, 3	NM_018283.3
OBSCN	37	NM_052843.3
PAG1	7	NM_018440.3
PAX5	2 - 5, 7 - 9	NM_016734.2
PDGFRA	10	NM_006206.5
PDGFRB	3, 18	NM_002609.3
PHF6	Full CDS	NM_032458
PLEKHG1	2	NM_001329801.1
PMS2	2, 9, 14	NM_000535.6
PRF1	3	NM_005041.5
PRKD1	6	NM_002742.2
PRKN	9	NM_004562.2
PRPS1	2 - 5, 7	NM_002764.3
PTEN	2, 5, 7	NM_000314.6
PTPN11	3, 4, 7, 8, 11, 13	NM_080601.2
PTPN14	10	NM_05401.4
RAG1	Full CDS	NM_000448
RAG2	Full CDS	NM_001243786
RANBP2	27	NM_006267.4
RB1	4, 8, 9, 13, 16, 19, 20, 23	NM_00321.2
RELN	29, 33, 40, 55, 59	NM_005045.3
RFPL4B	3	NM_001013734.2
RHOBTB2	3	NM_015178.2
RHOH	3	NM_004310.4
RIT1	5	NM_006912.5
RUNX1	3 - 9	NM_001754.4
SAE1	3	NM_005500.2
SBNO2	17, 20	NM_014963.2
SETD2	1 - 17, 19, 20	NM_014159.6
SF1	Full CDS	NM_201995
SF3A1	13	NM_005877.5

SH2B3	2 - 8	NM_005475.2
SLC25A6	4	NM_001636.3
SMAD5	8	NM_005903.6
SMARCA1	22	NM_003069.4
SOS1	6	NM_005633.3
SOX3	1	NM_005634.2
SP140	16	NM_007237.4
SPI1	3	NM_003120.2
SPRED1	3	NM_152594.2
STAG2	Full CDS	NM_001042749
STIM2	7	NM_020860.3
SUZ12	14, 15	NM_015355.3
SYNE1	14, 16, 35, 41, 62	NM_033071.3
TBL1XR1	5 - 9	NM_024665.5
TCF12	8	NM_207040.1
TCF3	Full CDS	NM_003200
TET2	Full CDS	NM_001127208
TP53	2 - 11	NM_000546.5
TRAPP	61	NM_003496.3
TRIM13	3	NM_052811.3
TYK2	8, 20, 22	NM_003331.4
WDR72	14	NM_182758.3
WEE1	5	NM_003390.3
WNK3	2	NM_020922.4
WT1	5 - 9	NM_00378.5
XPO1	15	NM_003400.3

Table S4. Chromosomic regions for fusion genes detection.

Fusion gene	Exon/Intron	Genomic position GRCh38/hg38
ETV6/*RUNX1* t(12;21)(p13;q22)	Intron 5 of *ETV6*	chr12: 11869929 - 11884409
BCR/*ABL1* t(9;22) (q34;q11)	Intron 1 of *BCR*	chr22: 23182170 - 23253663
BCR/ABL1 t(9;22)(p24;q11.2)	Exon 12-16 of *BCR*	chr22: 23288058 - 23295133
*MLL*r (*KMT2A*r)	Intron 9 to exon 12 of *MLL*	chr11: 118484173 - 118489862
TCF3/*PBX1* t(1;19)(q23;p13)and *TCF3*/*HLF* t(17;19)(q22;p13)	Intron 16 of *TCF3*	chr19: 1619457 - 1620958
CRLF2/*IGH* t(X;14)(p22; q32)	Upstream region of *CRLF2* exon 1	chrX: 1228511 - 1228820

Table S5. Chromosomes mapped to aneuploidy detection.

Aneuploidy	Chromosome
High hyperdiploidy	4
High hyperdiploidy	8
High hyperdiploidy	10
High hyperdiploidy	21
Low hypodiploidy	7
Low hypodiploidy	17

Table S6. Genes for CNVs detection.

ID gene	Exon	Transcript
IKZF1	Full CDS	NM_006060.6
CDKN2A	Full CDS	NM_000077.4
PAX5	2 - 9	NM_016734.2
ETV6	Full CDS	NM_001987
RB1	*4, 8, 9, 13, 16, 19, 20, 23*	NM_00321.2
BTG1	Full CDS	NM_001731.3
ERG	Full CDS	NM_004449.4

Table S7. Pharmacogenomic SNPs included.

ID gene	rs number or AA change
A2BP1	rs9924075
ABCB1	rs3770102, rs4728709, rs1128503, rs10264856
ABCC1	rs246240
ABCC2	rs3740065, rs3740066, rs717620
ABCC3	rs9895420
ABCC4	rs9516519, rs17268122, rs9556455
ACTG1	rs1135989
ADORA2A	rs2236624
APEX1	rs2307486
ARID5B	rs4948502, rs4948496, rs4948487, rs6479778, rs2893881, rs4948488, rs2393782, rs10821938, rs7923074, rs6479779, rs17215180
ATP6AP2	rs5917990
BCL2L11 (BIM)	rs724710
C3orf6	rs13358399
C5orf3, MFAP3	rs707184, rs1438588
CCDC24	rs368182
CDH12	rs10473594
CTLA-4	rs3087243, rs231775
CYP2C19	rs4244285, rs1057910
CYP3A5	rs776746
CYP4F2	rs2108622
DPYD	rs3918290
DROSHA	rs639174
FAM8A6P	rs1040637
FCHSD1	rs251177, rs6773449, rs6007758, rs41488548
FRMD4B	rs11707515, rs6549198
G6PD	rs5030868

GALNT10	rs11167667, rs12523441, rs7737215, rs10875583, rs6890748, rs6863455
GART	rs2070388
GATA3	rs3824662
GIT1	rs17808412
GNG2	rs12886319
GRIA1	rs2055083
	rs707176, rs17356099, rs13354399, rs10072570, rs11167640, rs6889909, rs4958676, rs6890057, rs10070447, rs4958351, rs4424038, rs7711124, rs7708391
HIVEP2, AIG1	rs200148
IMPDH1	rs4731448
ITPA	94C >A, rs41320251, rs1127354, rs7270101
KCNMA1	rs11001976, rs17480656, rs12765834, rs17389791, rs11001997
KIF13A	rs73726531
LOC642340	rs10170236
MAPK4	rs11662176, rs9953685
MCL1	rs3738485, 256G>C , 194T>G
MOCOS	rs3744900
MTHFR	rs786204016, rs1801131, rs1801133
MYRIP	rs17079534
NBN	1197A>G
NFATC2	rs6021191
NME1	rs2215290
NME1, NME2	rs3760467, rs1558254
NUDT15	rs746071566, rs766023281, 103A > G, rs116855232, rs186364861, rs147390019, rs554405994
P2RX1	rs17795186
PACSIN2	rs2413739
PAG1	rs877419
PDE4B	rs6683977, rs1402612, rs7578361, rs524770, rs641262, rs4265132, rs16965335, rs7141601, rs2613079,rs12751530
PTPRS	rs17763463, rs7600852

PYGL	rs7142143
RAD51AP2	rs424827, rs665312, rs7017705, rs13273490
SLC24A3	rs3748483
SLC25A37	rs2775139, rs2775134, rs1332944, rs2585498, rs2585499
SLC28A3	rs17428030, rs4588940, rs4305983, rs7043257, rs7035753, rs17087144
SLC36A3	rs7717132
SLCO1B1	rs11045879, rs4149081, rs4149056, rs2306283
SMARCB1	228G>T
SOD2	rs4880
TPMT	rs1800462, rs1800460, rs1142345, rs1800584, rs115106679, rs144041067, rs12201199, rs1142345, rs75543815, rs144041067, rs115106679
VKORC1	rs9923231
VSNL1	rs2710688
XDH	rs494852
XRCC1	26304C>T
XRCC3	rs1799794
ZP4	rs1565430

Table S8. Genes include in targeted TruSeqCustom Amplicon (TSCA) panel for ALL (pre-beta Test Plan for Illumina)

Genes			
ABL1	*EZH2*	*KRAS*	*RELN*
ADARB2	*FBXW7*	*LEF1*	*RUNX1*
ASMTL	*FLT3*	*MAPK1*	*SAE1*
BRAF	*GATA3*	*MYBL2*	*SETD2*
CDKN2A	*IDH1*	*NF1*	*SH2B3*
CREBBP	*IDH2*	*NOTCH1*	*SLC25A6*
CRLF2	*IKZF3*	*NR3C1*	*STAG2*
DNM2	*IL7R*	*NRAS*	*SUZ12*
DNMT3A	*JAK1*	*PAX5*	*TBL1XR1*
ECT2L	*JAK2*	*PHF6*	*TCF3*
EED	*JAK3*	*PTEN*	*TP53*
EP300	*KDM6A*	*PTPN11*	*WHSC1*
ETV6	*KMT2D*	*RB1*	*WT1*

Table S9. Oligonucleotide pair for fusion gene detection. Oligonucleotide pairs used in the PCR to confirm the presence of the fusion gene in the different samples. ID52, forward: intron 5 of *ETV6* (NM_001987.5) and reverse: intron 3 *RUNX1* (NM_001754.4). ID61, forward: intron 5 of *ETV6* (NM_001987.5) and reverse: intron 2 *RUNX1* (NM_001754.4). ID63, forward: intron 1 *BCR* (NM_004327.4) and reverse: intron 1 *ABL1* (NM_007313.2).

	Fusion gene	Forward 5′-3′	Reverse 5′-3′
**ID52**	*ETV6*/*RUNX1*	TGTGTGCAGCAGTACTTGACA (I.5 *ETV6*)	AAACGTTCTGGTTCTGCGGAT (I.3 *RUNX1*)
**ID61**	*ETV6*/*RUNX1*	TTCATGTAAAATAACCCTGGGG (I.5 *ETV6*)	GGCTCATATTTCAGCTCTAGAT (I.2 *RUNX1*)
**ID63**	*BCR*/*ABL1*	ATGTTGGTTCCACGTCCAAAC (I.1 *BCR*)	TGTTGCAGCATCCAGTTCATC (I.1 *ABL1*)

Table S10. List of variants detected in duplicate sequenced samples. VAF obtained in the sequencing of each of the samples (VAF1) and the one obtained the second time they were sequenced (VAF2).

Sample	*Gene*	**Exon, AA change & Transcript**	**VAF 1 (%)**	**VAF 2 (%)**
S1	*PAX5*	exon5:c.589_590insACTACCC:p.R197fs	37.66	37.55
S2	*PAX5*	exon6:c.748_749insCT:p.F250fs	39.89	35.69
	PAX5	exon3:c.T395C:p.V132A	10.99	6.86
	KCNE2	exon2:c.T170C:p.I57T	47.22	38.46
S3	*TCF3*	exon11:c.C923T:p.T308M	52.57	46.99
	NOTCH1	exon34:c.T7328G:p.V2443G	20.35	20.99

Table S11. Sensitivity and specificity of detection of different types of alterations. Sensitivity = true positives / (true positives + false negatives) x100 (%), specificity = true negatives / (true negatives + false positives) x100 (%).

**Type of alteration**	**Sensitivity** (%)	**Specificity** (%)
SNV/INDELS	96.3	90
CNVs	95.5	100
Aneuploidies	93.3	100
Fusion genes	89.7	100

Table S12. List of detected mutations. In the first column the sample identifier (ID), followed by the gene, VAF, function, exonic consequence, exon and AA change, cosmic82 identifier and avsnp144 identifier.

ID	Gene	VAF (%)	Function	Exonic consequence	Exon and AA change	cosmic82	avsnp144
ID1	*EP300*	49.25	exonic	nonsynonymous SNV	exon14:c.C2773A:p.P925T	COSM88779	rs148884710
	IKZF1	74.63	exonic	nonsynonymous SNV	exon5:c.G468C:p.Q156H	NA	NA
	PHF6	21.43	exonic	frameshift deletion	exon7:c.586_587del:p.R196fs	NA	NA
	PHF6	51.52	exonic	nonframeshift insertion	exon9:c.901_902insCGGGGG:p.Y301delinsSGD	NA	NA
ID2	*CBL*	69.58	exonic	nonsynonymous SNV	exon9:c.C1298T:p.P433L	COSM466951 9	rs140627020
	RFPL4B	65.1	exonic	nonsynonymous SNV	exon3:c.G679A:p.V227I	NA	NA
	TCF12	49.6	exonic	nonsynonymous SNV	exon8:c.T860C:p.V287A,TCF12	NA	NA
ID3	*IKZF3*	31.11	exonic	nonsynonymous SNV	exon8:c.G1516A:p.A506T	COSM597216 9	rs749495184
	SF1	45.17	exonic	nonsynonymous SNV	exon6:c.G582C:p.Q194H	NA	NA
	TCF3	42.73	exonic	nonsynonymous SNV	exon7:c.C472T:p.R158W	NA	rs749247091
ID5	*RUNX1*	40.76	exonic	nonsynonymous SNV	exon4:c.G320A:p.R107H	COSM438519 3	NA
ID8	*IKZF1*	17.18	exonic	frameshift insertion	exon7:c.732dupT:p.T244fs	NA	NA
ID9	*TET2*	50	exonic	nonsynonymous SNV	exon7:c.C3813G:p.C1271W	COSM120176	NA
ID10	*TET2*	35.6	exonic	nonsynonymous SNV	exon3:c.G1909A:p.E637K	NA	NA
ID11	*KMT2C*	41.61	exonic	nonsynonymous SNV	exon14:c.A2233G:p.I745V	NA	rs769543160
	NF1	19.67	exonic	frameshift insertion	exon18:c.2027dupC:p.T676fs	COSM123531 7	rs587781807
	NRAS	2.02	exonic	nonsynonymous SNV	exon2:c.G35T:p.G12V	COSM566	rs121913237

	NRAS	6.14	exonic	nonsynonymous SNV	exon2:c.G38A:p.G13D	COSM573	rs121434596
ID13	*RB1*	36.51	exonic	nonsynonymous SNV	exon9:c.C920T:p.T307I	NA	rs183898408
ID14	*CREBBP*	19.54	exonic	nonsynonymous SNV	exon30:c.T5050C:p.S1684P	NA	rs587783503
	NRAS	21.58	exonic	nonsynonymous SNV	exon2:c.G35A:p.G12D	COSM564	rs121913237
ID15	*NR3C2*	28.14	exonic	stopgain	exon2:c.C785G:p.S262X	NA	NA
ID16	*PAX5*	37.66	exonic	frameshift insertion	exon5:c.589_590insACTACCC:p.R197fs	NA	NA
ID18	*BRAF*	29.35	exonic	nonsynonymous SNV	exon15:c.G1780A:p.D594N	COSM27639	rs397516896
ID20	*NRAS*	4.49	exonic	nonsynonymous SNV	exon2:c.G35A:p.G12D	COSM564	rs121913237
ID21	*CDKN2A*	3.63	exonic	stopgain	exon2:c.C216A:p.C72X	COSM13567	NA
	KRAS	1.97	exonic	nonsynonymous SNV	exon4:c.A351T:p.K117N	COSM1562192	NA
	KRAS	16.87	exonic	nonsynonymous SNV	exon2:c.G35T:p.G12V	COSM1140133	rs121913529
	SLC25A6	41.01	exonic	nonsynonymous SNV	exon4:c.T856A:p.F286I	NA	NA
ID22	*CRLF2*	62.96	exonic	nonsynonymous SNV	exon5:c.C485A:p.S162Y	NA	NA
	NR3C1	48.75	exonic	nonsynonymous SNV	exon2:c.A695G:p.D232G	NA	NA
	NRAS	5.11	exonic	nonsynonymous SNV	exon2:c.G38A:p.G13D	COSM573	rs121434596
	TET2	44.56	exonic	nonsynonymous SNV	exon3:c.A2580T:p.Q860H	NA	NA
ID23	*FLT3*	12.86	exonic	nonsynonymous SNV	exon20:c.G2503T:p.D835Y	COSM783	rs121913488
	FLT3	41.24	exonic	nonsynonymous SNV	exon19:c.G2329A:p.E777K	NA	NA
	JAK3	41.18	exonic	nonsynonymous SNV	exon2:c.C23T:p.T8M	NA	rs145500023
	KRAS	14.68	exonic	nonsynonymous SNV	exon2:c.G35T:p.G12V	COSM1140133	rs121913529
ID25	*PAX5*	23.08	exonic	nonsynonymous SNV	exon3:c.T399A:p.S133R	NA	NA
ID26	*SH2B3*	51.58	exonic	nonsynonymous SNV	exon2:c.C464T:p.P155L	COSM1685385	rs531156627

ID27	*BCOR*	99.46	exonic	nonsynonymous SNV	exon4:c.G1780A:p.V594I	COSM5030822	rs764515953
	CDKN2A	56.94	exonic	frameshift insertion	exon2:c.225dupC:p.A76fs	NA	NA
	NRAS	6.79	exonic	nonsynonymous SNV	exon2:c.G35A:p.G12D	COSM564	rs121913237
	RANBP2	52.4	exonic	nonsynonymous SNV	exon27:c.T8800C:p.F2934L	NA	NA
ID28	*CDKN2A*	51.67	exonic	nonsynonymous SNV	exon2:c.G397A:p.A133T	NA	NA
	RUNX1	49.09	exonic	nonsynonymous SNV	exon8:c.G849C:p.Q283H	NA	NA
ID29	*NOTCH2*	50	exonic	nonsynonymous SNV	exon34:c.C6094A:p.H2032N	COSM1581340	rs143236410
	NRAS	5.13	exonic	nonsynonymous SNV	exon2:c.G35A:p.G12D	COSM564	rs121913237
ID30	*FLT3*	10.25	exonic	nonsynonymous SNV	exon20:c.A2504T:p.D835V	COSM784	rs121909646
ID31	*ASXL1*	45.98	exonic	nonsynonymous SNV	exon12:c.A2957G:p.N986S	COSM96383	rs145132837
	KRAS	39.7	exonic	nonsynonymous SNV	exon4:c.A351C:p.K117N	COSM1256061	rs770248150
	TET2	61.75	exonic	nonsynonymous SNV	exon3:c.G1285A:p.G429R	COSM5095018	rs201642693
ID32	*NRAS*	1.9	exonic	nonsynonymous SNV	exon2:c.G35C:p.G12A	COSM565	rs121913237
	NRAS	5.95	exonic	nonsynonymous SNV	exon2:c.G38A:p.G13D	COSM573	rs121434596
	PTPN11	33.76	exonic	nonsynonymous SNV	exon3:c.G226A:p.E76K	COSM13000	rs121918464
ID33	*KRAS*	5.85	exonic	nonsynonymous SNV	exon2:c.G38A:p.G13D	COSM1140132	rs112445441
ID34	*KRAS*	13.39	exonic	nonsynonymous SNV	exon2:c.G35T:p.G12V	COSM1140133	rs121913529
	PAX5	48.12	exonic	nonsynonymous SNV	exon9:c.T1032A:p.S344R	NA	NA
ID35	*DIS3*	20.49	splicing	NA	NA	NA	rs750580353
	NRAS	3.62	exonic	nonsynonymous SNV	exon3:c.C181A:p.Q61K	COSM580	rs121913254

ID36	*CREBBP*	68.71	exonic	nonsynonymous SNV	exon26:c.C4336T:p.R1446C	COSM88749	rs398124146
	IKZF1	7.77	exonic	nonsynonymous SNV	exon8:c.G1381C:p.V461L	NA	NA
	NRAS	10.2	exonic	nonsynonymous SNV	exon3:c.A183C:p.Q61H	COSM586	NA
	SETD2	74.13	exonic	stopgain	exon3:c.A1126T:p.K376X	NA	NA
ID37	*FLT3*	30.59	exonic	nonframeshift deletion	exon20:c.2508_2510del:p.836_837del	COSM19836	rs121913490
	PTPN11	2.96	exonic	nonsynonymous SNV	exon3:c.G226C:p.E76Q	COSM13016	rs121918464
ID38	*SH2B3*	48.11	exonic	nonsynonymous SNV	exon2:c.C639A:p.S213R	COSM168538 6	rs111360561
ID39	*ETV6*	19.07	exonic	nonsynonymous SNV	exon6:c.G1080C:p.W360C	NA	NA
	IRF8	48.95	exonic	nonsynonymous SNV	exon7:c.T895G:p.C299G	NA	NA
	KRAS	16.67	exonic	nonsynonymous SNV	exon4:c.G436A:p.A146T	COSM116519 8	rs121913527
	KRAS	21.86	exonic	nonsynonymous SNV	exon2:c.G35T:p.G12V	COSM114013 3	rs121913529
ID40	*ETV6*	35.19	exonic	frameshift deletion	exon7:c.1165_1166del:p.M389fs	NA	NA
	FLT3	36.51	exonic	nonsynonymous SNV	exon14:c.T1733C:p.M578T	COSM594529 7	NA
ID41	*PAX5*	44.13	exonic	nonsynonymous SNV	exon3:c.C239G:p.P80R	COSM85953	NA
	PAX5	50.66	exonic	nonsynonymous SNV	exon3:c.A215G:p.Y72C	COSM455949	NA
	PTPN11	4.86	exonic	nonsynonymous SNV	NM_080601:exon3:c.G181T:p.D61Y	COSM13011	NA
	PTPN11	30.87	exonic	nonsynonymous SNV	NM_080601:exon3:c.A227G:p.E76G	COSM13017	rs121918465
	IKZF1	41.93	exonic	frameshift insertion	exon3:c.97_98insTCGC:p.I33fs	NA	NA
ID42	*NF1*	69.35	exonic	stopgain	exon34:c.C4537T:p.R1513X	COSM24466	rs760703505
	SH2B3	49.28	exonic	nonsynonymous SNV	exon2:c.G622C:p.E208Q	COSM123539 0	rs202080221
	TP53	88.82	exonic	nonsynonymous SNV	exon6:c.G638A:p.R213Q	COSM131469	rs587778720

ID43	*ANKRD11*	26.24	exonic	nonsynonymous SNV	exon9:c.G6076A:p.A2026T	NA	rs752781169
	SETD2	27.67	exonic	nonsynonymous SNV	exon15:c.A6472G:p.N2158D	NA	NA
	TP53	42.27	splicing	NA	NA	COSM18655	NA
ID44	*EP300*	53.17	exonic	nonsynonymous SNV	exon14:c.C2773A:p.P925T	ICOSM88779	rs148884710
	IKZF1	6.42	exonic	nonsynonymous SNV	exon5:c.G472A:p.G158S	COSM303844	NA
	KMT2C	9.54	exonic	nonsynonymous SNV	exon18:c.G2926A:p.A976T	COSM3304323	rs779599464
	NRAS	35.68	exonic	nonsynonymous SNV	exon2:c.G35A:p.G12D	ICOSM564	rs121913237
	PAX5	35.29	exonic	nonsynonymous SNV	exon3:c.C224T:p.T75I	COSM4993960	NA
	SH2B3	38.41	exonic	stopgain	exon6:c.1200_1201insTAGGGGT:p.E400_Y401delinsEX	NA	NA
ID45	*ABL2*	46.67	exonic	nonsynonymous SNV	exon12:c.A1945T:p.T649S	NA	rs141450341
	NRAS	39.82	exonic	nonsynonymous SNV	exon2:c.G38A:p.G13D	COSM573	rs121434596
ID46	*EP300*	51.46	exonic	nonsynonymous SNV	exon14:c.C2773A:p.P925T	COSM88779	rs148884710
	IKZF1	10	exonic	nonsynonymous SNV	exon5:c.G472A:p.G158S	COSM303844	NA
ID47	*ETV6*	41.37	exonic	frameshift insertion	exon5:c.479_480insGA:p.R160fs	NA	NA
	JAK2	6.74	exonic	nonsynonymous SNV	exon16:c.A2044T:p.I682F	COSM303887	NA
	JAK2	10.06	exonic	nonsynonymous SNV	exon20:c.G2617A:p.D873N	COSM303882	NA
	NRAS	12.27	exonic	nonsynonymous SNV	exon3:c.C181A:p.Q61K	COSM580	rs121913254
	NRAS	16.22	exonic	nonsynonymous SNV	exon3:c.A182T:p.Q61L	COSM583	rs11554290
ID48	*JAK2*	3.04	exonic	nonsynonymous SNV	exon16:c.A2049T:p.R683S	COSM29302	NA
	JAK2	12.13	exonic	nonsynonymous SNV	exon16:c.T2081C:p.F694S	NA	NA
	JAK2	29.87	exonic	nonsynonymous SNV	exon16:c.A2047G:p.R683G	COSM29300	NA
	NRAS	4.22	exonic	nonsynonymous SNV	exon2:c.G35A:p.G12D	COSM564	rs121913237

ID49	*JAK2*	25.97	exonic	nonsynonymous SNV	exon20:c.C2624A:p.T875N	COSM23940	NA
	MPL	42.68	exonic	nonsynonymous SNV	exon9:c.T1432A:p.S478T	NA	rs781129632
ID51	*NRAS*	7.58	exonic	nonsynonymous SNV	exon2:c.G35A:p.G12D	COSM564	rs121913237
ID52	*KRAS*	9.02	exonic	nonsynonymous SNV	exon2:c.G34C:p.G12R	COSM518	rs121913530
ID53	*PEAK1*	53.49	exonic	nonsynonymous SNV	exon5:c.A2689G:p.T897A	NA	NA
ID54	*FAT1*	49.12	exonic	nonsynonymous SNV	exon2:c.G1555A:p.V519M	NA	NA
ID55	*ASXL1*	48.55	exonic	nonsynonymous SNV	exon12:c.G1831A:p.A611T	COSM288926<br>3	rs372418554
	KMT2C	10.71	exonic	nonsynonymous SNV	exon18:c.G2914A:p.G972R	NA	rs746739227
	NRAS	21.83	exonic	nonsynonymous SNV	exon2:c.G38A:p.G13D	COSM573	rs121434596
ID57	*NRAS*	45.12	exonic	nonsynonymous SNV	exon2:c.G35A:p.G12D	COSM564	rs121913237
	XPO1	51.52	exonic	nonsynonymous SNV	exon15:c.G1711A:p.E571K	COSM96797	NA
ID58	*SPI1*	31.92	exonic	frameshift insertion	exon3:c.186_187insTCCCTCC:p.E63fs	NA	NA
ID59	*KRAS*	23.36	exonic	nonsynonymous SNV	exon2:c.G34A:p.G12S	COSM115250<br>6	rs121913530
	NRAS	8.03	exonic	nonsynonymous SNV	exon2:c.G35A:p.G12D	COSM564	rs121913237
	PTPN11	4.93	exonic	nonsynonymous SNV	NM_080601:exon3:c.C215A:p.A72D	COSM13035	NA
ID60	*CRLF2*	2.84	exonic	nonsynonymous SNV	exon6:c.T695G:p.F232C	COSM41268	NA
	DNMT3A	2.91	exonic	nonsynonymous SNV	exon23:c.C2711T:p.P904L	COSM87007	rs149095705
	JAK2	6.67	exonic	nonsynonymous SNV	exon16:c.A2047G:p.R683G	COSM29300	NA
	JAK2	16.85	exonic	nonsynonymous SNV	exon20:c.C2624A:p.T875N	COSM23940	NA
ID61	*EZH2*	3.2	exonic	nonsynonymous SNV	exon17:c.C1948T:p.H650Y	COSM53040	rs193921147
	SETD2	5.11	exonic	nonsynonymous SNV	exon3:c.G1369A:p.E457K	NA	NA
	SETD2	5.17	exonic	frameshift insertion	exon3:c.1367dupG:p.R456fs	NA	NA
	SETD2	18.45	exonic	stopgain	exon3:c.C995G:p.S332X	NA	NA

	SETD2	19.49	exonic	nonsynonymous SNV	exon3:c.C805G:p.Q269E	COSM5986197	NA
ID63	*ZNF384*	44.84	exonic	nonsynonymous SNV	exon8:c.T1007A:p.L336H	NA	NA
ID64	*FLT3*	43.79	exonic	nonsynonymous SNV	exon5:c.G580A:p.V194M	COSM28039	rs146030737
ID65	*CSF2RA*	25	splicing	NA	NA	NA	NA
	IL7R	25.71	exonic	nonsynonymous SNV	exon3:c.G291T:p.K97N	COSM1067537	NA
	NRAS	3.64	exonic	nonsynonymous SNV	exon2:c.G35A:p.G12D	COSM564	rs121913237
ID66	*COG1*	37.93	exonic	nonsynonymous SNV	exon7:c.A1741G:p.I581V	NA	rs757072904
ID67	*NRAS*	26.62	exonic	nonsynonymous SNV	exon2:c.G38A:p.G13D	COSM573	rs121434596
	PTPN11	2.24	exonic	nonsynonymous SNV	exon3:c.T211C:p.F71L	COSM13039	rs397507512
ID68	*CREBBP*	42.81	exonic	nonsynonymous SNV	exon31:c.A5617G:p.N1873D	NA	NA
	ETV6	35.39	exonic	nonsynonymous SNV	exon7:c.G1223C:p.R408T	NA	NA
	NRAS	31.07	exonic	nonsynonymous SNV	exon2:c.G38A:p.G13D	COSM573	rs121434596
	RAG1	31.27	exonic	nonframeshift insertion	exon2:c.775_776insGCT:p.S259delinsSC	NA	NA
ID69	*KMT2D*	50	exonic	nonsynonymous SNV	exon11:c.C3793T:p.P1265S	NA	NA
	NOTCH1	48.37	exonic	nonsynonymous SNV	exon34:c.G6392A:p.G2131D	NA	NA
	PAX5	33.51	exonic	stopgain	exon5:c.601_602insCTT:p.E201delinsAX	NA	NA
	PAX5	36.04	exonic	nonsynonymous SNV	exon5:c.G601C:p.E201Q	NA	NA
ID70	*JAK2*	15.84	exonic	nonsynonymous SNV	exon16:c.A2049T:p.R683S	COSM29302	NA
ID71	*GRM1*	42	exonic	nonsynonymous SNV	exon8:c.C2756T:p.T919I	NA	rs536808733
	PHF6	18.42	exonic	stopgain	exon5:c.C385T:p.R129X	COSM4775093	NA
	RUNX1	48.68	exonic	nonsynonymous SNV	exon4:c.G136A:p.A46T	NA	NA
ID72	*EP300*	42.98	exonic	nonsynonymous SNV	exon14:c.C2773A:p.P925T	COSM88779	rs148884710
	NRAS	3.97	exonic	nonsynonymous SNV	exon2:c.G35A:p.G12D	COSM564	rs121913237

ID73	*CRLF2*	25.69	exonic	nonsynonymous SNV	exon6:c.T695G:p.F232C	COSM41268	NA
	JAK2	14.96	exonic	nonsynonymous SNV	exon16:c.A2049T:p.R683S	COSM29302	NA
	PAX5	17.27	exonic	frameshift insertion	exon6:c.621_622insCTGGCGCCTTGGCCTG:p.V208fs	NA	NA
ID74	*JAK2*	41.1	exonic	nonsynonymous SNV	exon16:c.A2047G:p.R683G	COSM29300	NA
ID75	*NRAS*	34	exonic	nonsynonymous SNV	exon2:c.G34A:p.G12S	COSM563	rs121913250

Table S13. Mutations described in REH cell line. The following table shows a number of mutations detected in the REH cell line and two clones established from it. In the first column the gene, altered nucleotide (CDS mutation), altered amino acid (AA mutation) and variant allele frequency (VAF) detected in REH cells, clon 1 and clon 2.

**Gene**	**CDS mutation**	**AA mutation**	**VAF (%)**		
			**REH**	**Clon 1**	**Clon 2**
TBLX1R1	c.G497A	p.R166Q	53.3	62.36	48.94
IKZF1	c.G1079A	p.R360H	44.14	46.96	49.76
NOTCH1	c.C7568T	p.S2523L	26	0	0
IL27	c.G100A	p.G34R	32.32	29.9	34.43
TP53	c.C541T	p.R181C	29.3	0	0
**GATA3**	c.C953T	p.A318V	41.85	47.19	50.78
BCL11B	c.G1251A	p.T417T	40.71	44.01	42.41
BCL11B	c.C915T	p.F305F	52.26	46.63	45.93

Table S14. Frequency of pharmacogenetic SNPs detected in the patient cohort.

rs number	Gene	Heterozygosis frequency (%)	Homozygosis frequency (%)	Total frequency (%)
**rs1128503**	*ABCB1*	18.7	8.0	29.3
**rs4728709**	*ABCB1*	1.3	0.0	1.3
**rs3740066**	*ABCC2*	17.3	2.7	20.0
**rs3740065**	*ABCC2*	13.3	0.0	13.3
**rs717620**	*ABCC2*	12.0	0.0	12.0
**rs17216310**	*ABCC2*	1.3	0.0	1.3
**rs4793665**	*ABCC3*	21.3	10.7	32.0
**rs9895420**	*ABCC3*	4.0	1.3	5.3
**rs9516519**	*ABCC4*	6.7	0.0	6.7
**rs9556455**	*ABCC4*	4.0	1.3	5.3
**rs1139405**	*ACTG1*	13.3	20.0	33.3
**rs1135989**	*ACTG1*	16.0	5.3	21.3
**rs1048945**	*APEX1*	6.7	0.0	6.7
**rs2307486**	*APEX1*	1.3	0.0	1.3
**rs10821936**	*ARID5B*	40.0	18.7	58.7
**rs7073837**	*ARID5B*	36.0	18.7	54.7
**rs10994982**	*ARID5B*	38.7	10.7	49.3
**rs10740055**	*ARID5B*	37.3	10.7	48.0
**rs10821935**	*ARID5B*	36.0	10.7	46.7
**rs7089424**	*ARID5B*	38.7	5.3	44.0
**rs6479778**	*ARID5B*	12.0	22.7	34.7
**rs4614389**	*ARID5B*	12.0	22.7	34.7
**rs2893881**	*ARID5B*	12.0	22.7	34.7
**rs2393782**	*ARID5B*	13.3	18.7	32.0
**rs4948496**	*ARID5B*	22.7	8.0	30.7
**rs7923074**	*ARID5B*	14.7	14.7	29.3
**rs10821938**	*ARID5B*	14.7	14.7	29.3
**rs4948488**	*ARID5B*	12.0	16.0	28.0
**rs17215180**	*ARID5B*	21.3	5.3	26.7
**rs6479779**	*ARID5B*	16.0	9.3	25.3
**rs4948502**	*ARID5B*	16.0	4.0	20.0
**rs7075591**	*ARID5B*	14.7	4.0	18.7
**rs4948487**	*ARID5B*	12.0	4.0	16.0

**rs2393783**	*ARID5B*	8.0	6.7	14.7
**rs77918077**	*ARID5B*	5.3	0.0	5.3
**rs10994973**	*ARID5B*	2.7	0.0	2.7
**rs77708105**	*ARID5B*	1.3	0.0	1.3
**rs188676594**	*ARID5B*	1.3	0.0	1.3
**rs149113357**	*ARID5B*	1.3	0.0	1.3
**rs776746**	*CYP3A5*	4.0	26.7	30.7
**rs2108622**	*CYP4F2*	13.3	4.0	17.3
**rs17376848**	*DPYD*	2.7	0.0	2.7
**rs3918290**	*DPYD*	1.3	0.0	1.3
**rs3918289**	*DPYD*	1.3	0.0	1.3
**rs639174**	*DROSHA*	1.3	0.0	1.3
**rs251177**	*FCHSD1*	18.7	0.0	18.7
**rs11707515**	*FRMD4B*	9.3	1.3	10.7
**rs7737215**	*GALNT10*	16.0	12.0	28.0
**rs6890748**	*GALNT10*	17.3	9.3	26.7
**rs12523441**	*GALNT10*	13.3	9.3	22.7
**rs11167667**	*GALNT10*	13.3	8.0	21.3
**rs10875583**	*GALNT10*	13.3	8.0	21.3
**rs153440**	*GALNT10*	8.0	0.0	8.0
**rs139021674**	*GALNT10*	1.3	0.0	1.3
**rs422628**	*GATA3*	34.7	61.3	96.0
**rs3839918**	*GATA3*	20.0	40.0	60.0
**rs1058240**	*GATA3*	20.0	40.0	60.0
**rs9746**	*GATA3*	22.7	4.0	26.7
**rs3824662**	*GATA3*	17.3	5.3	22.7
**rs11567941**	*GATA3*	9.3	1.3	10.7
**rs552157419**	*GATA3*	1.3	0.0	1.3
**rs12886319**	*GNG2*	9.3	24.0	33.3
**rs707176**	*GRIA1*	16.0	2.7	18.7
**rs6889794**	*GRIA1*	12.0	5.3	17.3
**rs4958351**	*GRIA1*	13.3	0.0	13.3
**rs10070447**	*GRIA1*	13.3	0.0	13.3
**rs6890057**	*GRIA1*	10.7	0.0	10.7
**rs6889909**	*GRIA1*	10.7	0.0	10.7
**rs4958676**	*GRIA1*	10.7	0.0	10.7
**rs13354399**	*GRIA1*	10.7	0.0	10.7

**rs10072570**	*GRIA1*	10.7	0.0	10.7
**rs7711124**	*GRIA1*	9.3	0.0	9.3
**rs4424038**	*GRIA1*	8.0	1.3	9.3
**rs7708391**	*GRIA1*	8.0	0.0	8.0
**rs67708322**	*GRIA1*	6.7	0.0	6.7
**rs2055083**	*GRIA1*	5.3	1.3	6.7
**rs17356099**	*GRIA1*	5.3	0.0	5.3
**rs11167640**	*GRIA1*	4.0	0.0	4.0
**rs11743325**	*GRIA1*	2.7	0.0	2.7
**rs72804610**	*GRIA1*	1.3	0.0	1.3
**rs10954184**	*IMPDH1*	40.0	17.3	57.3
**rs10954183**	*IMPDH1*	37.3	17.3	54.7
**rs4731448**	*IMPDH1*	9.3	21.3	30.7
**rs7270101**	*ITPA*	10.7	1.3	12.0
**rs1127354**	*ITPA*	5.3	0.0	5.3
**rs10762752**	*KCNMA1*	44.0	4.0	48.0
**rs17480264**	*KCNMA1*	9.3	0.0	9.3
**rs17480656**	*KCNMA1*	6.7	0.0	6.7
**rs17389791**	*KCNMA1*	6.7	0.0	6.7
**rs12765834**	*KCNMA1*	6.7	0.0	6.7
**rs11001997**	*KCNMA1*	6.7	0.0	6.7
**rs11001976**	*KCNMA1*	6.7	0.0	6.7
**rs11662176**	*MAPK4*	13.3	2.7	16.0
**rs9953685**	*MAPK4*	13.3	1.3	14.7
**rs3738485**	*MCL1*	14.7	10.7	25.3
**rs34645101**	*MCL1*	1.3	0.0	1.3
**rs376254012**	*MOCOS*	1.3	0.0	1.3
**rs3744900**	*MOCOS*	1.3	0.0	1.3
**rs4846051**	*MTHFR*	1.3	34.7	36.0
**rs1801133**	*MTHFR*	20.0	2.7	22.7
**rs1801131**	*MTHFR*	14.7	2.7	17.3
**rs709816**	*NBN*	20.0	0.0	20.0
**rs34767364**	*NBN*	4.0	0.0	4.0
**rs61973267**	*NUDT15*	9.3	0.0	9.3
**rs138959770**	*NUDT15*	1.3	0.0	1.3
**rs2413739**	*PACSIN2*	18.7	5.3	24.0
**rs117008677**	*PACSIN2*	1.3	1.3	1.3

**rs7006101**	*PAG1*	13.3	1.3	14.7
**rs1866275**	*PAG1*	1.3	0.0	1.3
**rs140643833**	*PAG1*	1.3	0.0	1.3
**rs641262**	*PDE4B*	12.0	13.3	25.3
**rs524770**	*PDE4B*	9.3	16.0	25.3
**rs12137115**	*PDE4B*	10.7	13.3	24.0
**rs12137080**	*PDE4B*	10.7	2.7	13.3
**rs71121613**	*PYGL*	10.7	5.3	16.0
**rs882860**	*PYGL*	12.0	1.3	13.3
**rs665312**	*RAD51AP2*	9.3	1.3	10.7
**rs7043257**	*SLC28A3*	12.0	21.3	33.3
**rs7035753**	*SLC28A3*	13.3	18.7	32.0
**rs4588940**	*SLC28A3*	18.7	12.0	30.7
**rs17087144**	*SLC28A3*	14.7	5.3	20.0
**rs17428030**	*SLC28A3*	4.0	1.3	5.3
**rs75316220**	*SLC28A3*	2.7	0.0	2.7
**rs4149057**	*SLCO1B1*	17.3	9.3	25.3
**rs4149056**	*SLCO1B1*	13.3	1.3	14.7
**rs11045879**	*SLCO1B1*	12.0	1.3	13.3
**rs4149081**	*SLCO1B1*	4.0	0.0	4.0
**rs4880**	*SOD2*	21.3	6.7	28.0
**rs2842949**	*TPMT*	8.0	8.0	16.0
**rs2842934**	*TPMT*	2.7	1.3	4.0
**rs12201199**	*TPMT*	1.3	0.0	1.3
**rs494852**	*XDH*	4.0	4.0	8.0
**rs1799782**	*XRCC1*	5.3	0.0	5.3
**rs2307170**	*XRCC1*	1.3	0.0	1.3
**rs1799779**	*XRCC1*	1.3	0.0	1.3
**rs861529**	*XRCC3*	2.7	33.3	36.0
**rs1799794**	*XRCC3*	14.7	2.7	17.3

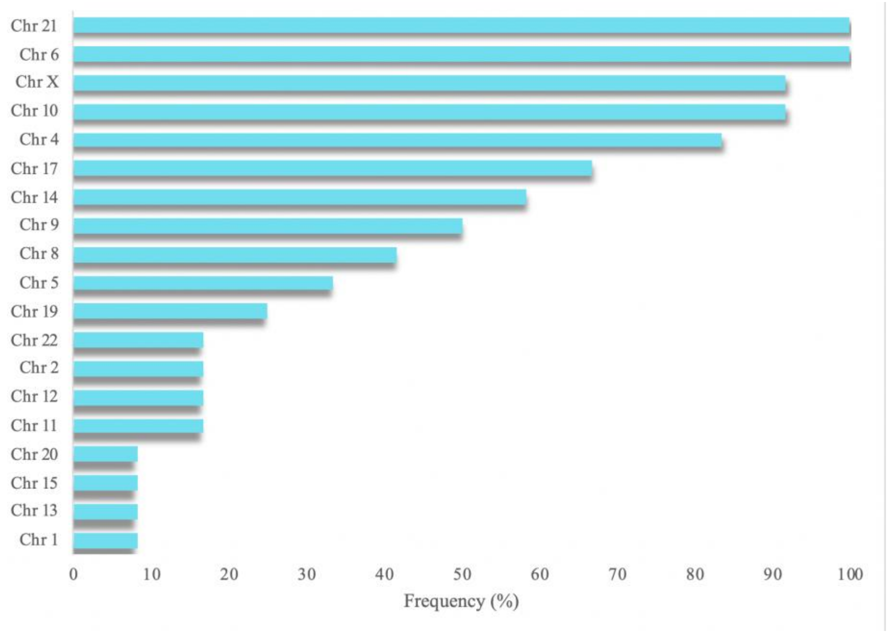

Figure S1. Frequency of trisomies in high hyperdiploidy cases. The following figure shows the frequency of trisomies observed in high hyperdiploidy patients included in this study in decreasing order.

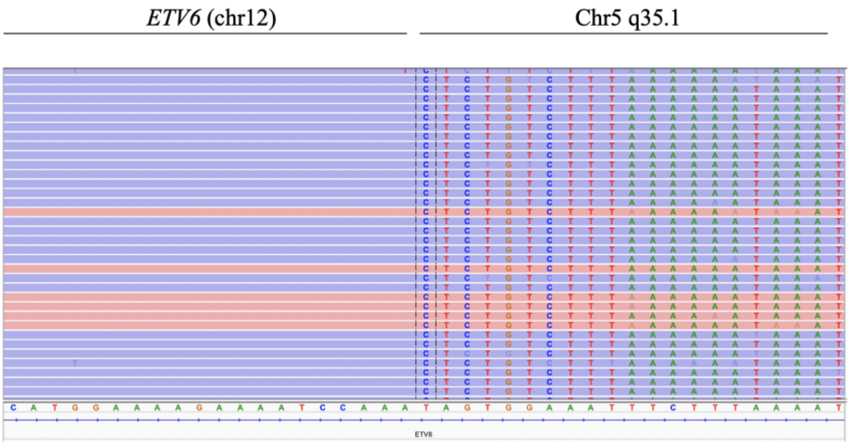

Figure S2. t(5;12)(q35;p13) visualized with IGV. t(5;12)(q35;p13) observed in (sample ID13). In the left region the reads aligned with intron 5 of *ETV6* and to the right the reads aligned with chromosome 5 q35.1.
